# Commonness and ecology, but not bigger brains, predict urban living in birds

**DOI:** 10.1186/s12898-015-0044-x

**Published:** 2015-04-11

**Authors:** Svein Dale, Jan T Lifjeld, Melissah Rowe

**Affiliations:** Department of Ecology and Natural Resource Management, Norwegian University of Life Sciences, P.O. Box 5003, NO-1432 Ås Oslo, Norway; Natural History Museum, University of Oslo, P.O. Box 1172 Blindern, NO-0318 Oslo, Norway

**Keywords:** Bird communities, Colonization pressure, Brain size, Source population, Urban ecology

## Abstract

**Background:**

Several life history and ecological variables have been reported to affect the likelihood of species becoming urbanized. Recently, studies have also focused on the role of brain size in explaining ability to adapt to urban environments. In contrast, however, little is known about the effect of colonization pressure from surrounding areas, which may confound conclusions about what makes a species urban. We recorded presence/absence data for birds in 93 urban sites in Oslo (Norway) and compared these with species lists generated from 137 forest and 51 farmland sites surrounding Oslo which may represent source populations for colonization.

**Results:**

We found that the frequency (proportion of sites where present) of a species within the city was strongly and positively associated with its frequency in sites surrounding the city, as were both species breeding habitat and nest site location. In contrast, there were generally no significant effects of relative brain mass or migration on urban occupancy. Furthermore, analyses of previously published data showed that urban density of birds in six other European cities was also positively and significantly associated with density in areas outside cities, whereas relative brain mass showed no such relationship.

**Conclusions:**

These results suggest that urban bird communities are primarily determined by how frequently species occurred in the surrounding landscapes and by features of ecology (i.e. breeding habitat and nest site location), whereas species’ relative brain mass had no significant effects.

**Electronic supplementary material:**

The online version of this article (doi:10.1186/s12898-015-0044-x) contains supplementary material, which is available to authorized users.

## Background

Humans dominate increasingly large parts of the Earth [1], and understanding what determines the ability of wildlife to exploit urbanized areas is important for biodiversity conservation [2-4]. Studies have indicated that life history and ecological variables, such as broad environmental tolerance (i.e. niche breadth), omnivory, safe nest sites, non-migratory habits and high fecundity, increase the likelihood that bird species will occur in urban environments [5-9]. Several of these characteristics overlap with those found to affect invasion success of introduced species [10-12]. In birds, recent studies have also suggested that relatively large brains predispose species for urban living [13,14], similar to the effect of brain size on invasion success [11,15]. This may be because brain size is related to feeding innovations and behavioral flexibility (e.g. [16]) which may promote invasion success and adaptability. Larger brains may therefore help birds to exploit new food resources, and avoid novel predators and human disturbance [17]. However, several studies have failed to find a relationship between brain size and urban living [18-20], and it has been questioned whether whole brain size is in fact a useful measure of behavioural flexibility and innovation [21].

Typically, urban areas are inhabited by a limited number of species that represent a subset of the regional species pool [3,5,9,22]. It has been suggested that urbanization depends on high population density in the original habitat and good dispersal ability [23], which can be considered a specific case of the idea that communities may be assembled by random dispersal [24,25]. Wildlife in the surroundings of urban areas may act as source populations and ‘seed’ urban populations similar to a propagule pressure in biological invasions [26,27]. Thus, urban bird communities might reflect the regional bird community through immigration from exurban source populations. To date, however, the possible role of source population size or relative commonness of species in determining urban bird communities has not received much attention, instead the dominating view has been that urban areas favour a small set of species which have particular traits making them able to adapt to novel conditions (see [28] and references therein).

While many studies have gathered data on species’ occurrence both in urban and surrounding areas (see e.g. review in [29]), explicit analyses of quantitative data to compare species occurrence in urban areas with occurrence in surrounding areas are relatively limited. Moreover, of the few studies that do address this specific issue, many have used national or other large-scale indices of population size in analyses of urbanization [8,9], which overlooks potentially important spatial variation in source populations [30]. Møller et al. reported that urban species were those with high population densities in their ancestral rural habitats [31]. More recently, Sol et al. showed that the abundance of avian species in urbanised environments was positively correlated with the relative abundance of species in the surroundings [20]. In contrast, other studies have concluded that there were no relationships between bird densities in urban and surrounding rural areas [32], or that species richness of urban communities were independent of the diversity in adjacent landscapes [29]. Consequently, further large scale analyses examining the association between species occurrence in urban sites and the surrounding rural areas are needed to help clarify the impact of adjacent landscapes in determining urban communities and to allow us to determine how generalizable this process (i.e. urban community assemblage via random dispersal) may be.

Importantly, if urban bird communities reflect species occurrence in the surrounding environment, analyses of relationships between life history or ecological factors (e.g. fecundity, niche breadth, nest sites etc.) and urban success need to control for source population size in order to avoid spurious correlations. For example, certain life history and ecological factors may have caused species to become common in non-urban habitats, but may have no effect *per se* in promoting invasion of urban areas. Bonier and coworkers [6] found that urban species had broader environmental tolerance than rural congeners, but because the potential impact of source population size on urban occurrence was not assessed the possibility that species may have become urban simply because they occurred more frequently in the adjacent landscape cannot be ruled out. Similarly, Maklakov and coworkers [14] claimed that relatively large brain size predisposes species for urban establishment, but emphasized that their analyses ignored variation owing to ecological factors. Moreover, that study did not incorporate information on potential source population size. Thus, in line with previous studies [19,20], we suggest that investigations of urban bird community composition need to concurrently assess the effects of rural source population size, relative brain size and relevant ecological traits in order to more fully understand the importance of these traits for species adaptation to urban life.

Here, using phylogenetically controlled analyses, we investigated the relative importance of three potential predictors of urban bird communities: source population size, relative brain mass and ecology. More specifically, using data on avian species from Oslo (Norway), we tested for an association between species occurrence (presence/absence) in urban sites and species occurrence in surrounding rural sites, relative brain mass and three key features of a species ecology that are thought to influence relative brain mass or the way species interact with their environment, i.e. migratory status, breeding habitat and nesting site. Furthermore, we also used previously published data to analyse the relative importance of two of these predictors (i.e. rural population density and relative brain mass) on urban population density for an additional six cities across Europe.

## Methods

### Urban sites

Birds were censused in 93 parks, cemeteries and other urban green spaces in Oslo (~60°N, 11°E, Additional file 1: Table S1, Additional file 2: Figure S1). This represented nearly all urban green spaces larger than 1 ha in built-up areas of Oslo. Urban sites had a median size of 8.6 ha (range 0.6 - 98.1 ha), and vegetation varied from intensively managed parks with ornamental deciduous trees and lawns, to green spaces with a mix of managed parkland and patches of more or less natural vegetation. In these sites, natural vegetation is dominated by deciduous forest and mixed forest; pure coniferous forest occurs predominantly outside built-up areas.

Downtown Oslo is a predominantly commercial area and green spaces are mostly restricted to small parks and cemeteries. Birds are also restricted to such sites in the most urbanized part of Oslo, except for a handful of species (see further in Discussion). From central Oslo there is a gradient through areas dominated by apartment buildings to residential areas with a larger amount of vegetation outside parks and other urban green spaces. The residential areas are adjacent to continuous forest (mostly boreal forest dominated by conifers) along much of the periphery of the city, but in some areas residential areas are adjacent to farmland. From the central part of Oslo distances to closest areas of continuous boreal forest are typically 5–6 km. During the study period, Oslo had 520,000-550,000 inhabitants representing an average population density of > 3,500 persons/km^2^.

Each urban site was censused (by SD) a total of three times, giving a total of 279 individual censuses. Censuses were conducted between sunrise and midday during the breeding season (mainly May-June) in 2003–2007. Each site was censused in at least two different years, at three different times of the breeding season (early, middle and late), and at different times of the day. Censuses consisted of walking slowly through each site, and paths were chosen to cover each site equally well and such that no part of the site was more than 100 m away from the path used. Censuses lasted 10–55 min and increased with the size of the site. Species were recorded as present or absent for each site, based on visual and vocal observations from all three censuses. As censuses aimed to detect potential breeding land bird species, wetland and passage migrant species (i.e. those migrating through and not breeding in the city) were excluded. Urban occurrence was measured as proportion of sites used for each species.

We chose to record species as present/absent instead of estimating density in urban areas because data for rural sites had been collected as species presence/absence and because methods used for obtaining density estimates (i.e. line transects, point counts) do not permit sampling of the full area of each site, which we considered necessary for a complete overview of the urban bird community. Moreover, collecting presence/absence data is considered an efficient method for large-scale monitoring [33]. It should also be noted that our index of urbanization (proportion of sites used for each species) gives a comprehensive, continuous measure, whereas several previous studies have simply compared urbanized versus non-urbanized (or less urbanized) species [6,8,9,14,18]. Finally, occurrence frequency and population density are likely to be positively correlated because widespread species generally have higher densities [32,34,35] and a link between presence/absence data (occupancy) and abundance is also expected on theoretical grounds [33]. This was also the case in our urban data set where occurrence frequency was significantly correlated with abundance based on approximate numbers of individuals observed during censuses (using highest count from the three censuses of each site; total number of birds observed across all sites: *r*_*s*_ = 0.97, *N* = 60 species, *P* < 0.0001, mean number observed per occupied site: *r*_*s*_ = 0.83, *N* = 60 species, *P* < 0.0001). Note that throughout the manuscript, we use the term commonness broadly, and as such this term encompasses both occurence frequency (occupancy) and population density.

### Sites surrounding the city

Data on species presence/absence in sites surrounding the city (i.e. rural sites) were taken from species lists generated for > 1500 sites in Oslo and the neighbouring county of Akershus during fieldwork conducted for biodiversity conservation purposes (primarily by SD) during 1995–2011 (see e.g. [36]). Sites were selected to provide representative sampling of different habitats and elevations and encompass spatial variation. Sites were generally defined according to topographical and spatial features (such as hills, valleys, patches of farmland). From this extensive dataset, we selected all forest and farmland sites located within Oslo county (but outside the city itself) and the three closest municipalities in Akershus county (Bærum, Lørenskog and Nittedal) that had been investigated thoroughly at least once during the breeding season. Thus, 137 forest and 51 farmland sites served as potential source areas for land bird species found within urban sites in Oslo city. The larger number of forest sites than farmland sites reflected that forests dominate the surroundings of Oslo (see further in Additional file 3). Twelve sites had both forest and farmland, thus there were 176 different source sites in total. Typically, sites ranged from 50–500 ha in total area (median 110 ha, range 11–1780 ha), and the midpoint of source sites had a median distance of 7.0 km from closest built-up areas of Oslo (range 0.2-21.8 km). Only observations made within the breeding season (mainly May-June, though also April and July if observations clearly suggested breeding behaviour and excluded the possibility of passage migrants or post-breeding movements) were used to calculate frequency of occurrence across sites, and, as before, wetland and passage migrant (i.e. non-breeding) species were excluded. For sites visited only once, surveys lasted 1–5 hours, depending on the size of each site, and were conducted from sunrise until midday. Census paths were chosen to cover habitat diversity within sites in order to detect a high proportion of species present. In general, forest and farmland sites had lower survey effort per unit of area relative to urban sites, although time spent surveying was usually longer. See further in Additional file 3 for evaluations of the comparability of data from rural and urban sites.

### Relative brain mass

We examined relative brain mass by including both brain mass and body mass (log-transformed) as independent variables in our statistical models. This approach controls for allometry between brain mass and body mass, and is preferable to the use of the simple residuals from a regression between the two variables (e.g. [37-39]) or expressing relative brain mass as a ratio of the two variables (e.g. [40,41]). Furthermore, by including both brain and body mass as independent variables in models it is possible to investigate additional body mass effects that are independent of brain mass. Finally, this approach is commonly used in studies of relative brain size (e.g. [14,19]) and as a method for normalising data for variation in body size in a range of traits (e.g. metabolic rate [42], testes size [43,44]).

Data on body mass and brain mass were taken from the literature. Body mass data was specific to populations in Norway [45]. In contrast, data on whole brain mass specific to Norway were unavailable, thus data were taken from European sources [46-52]. More specifically, we combined data from all sources to provide a complete list for all species in our study (see Additional file 1: Table S2 for details). Moreover, brain mass was strongly correlated among the five sources (*r*_*s*_ = 0.97-1.00 in all ten comparisons, *N* = 36–56, all *P* < 0.0001). Similarly, body mass data given by the five sources for brain mass were strongly correlated with the Norwegian body mass data [45] (*r*_*s*_ = 0.99-1.00, *N* = 49–82, all *P* < 0.0001). Values of body and brain mass are provided in Additional file 1: Table S2).

### Ecological variables

Although a broad range of life history and ecological factors have been linked to urban bird community composition (e.g. diet, nesting site, fecundity, etc. [5-9,20]), we focused on three key features of a species ecology that are thought to influence either relative brain mass or the way species interact with their environment. First, migratory status (i.e. resident vs. migratory) was investigated because this factor appears to have a substantial effect on brain mass [52]. Species were classified as resident or migratory based on literature relevant to local conditions [53]; species in which a minor part of the population is resident were coded as migratory.

Next, the likelihood of species finding suitable breeding sites within the urban sites is expected to influence how frequently they are found in such urbanized areas. Therefore, we also investigated the effects of species breeding habitat. Species breeding habitat was classified following Dale et al. [53]; the major reference work on the status and distribution of birds in Oslo and Akershus counties. More specifically, species were classified into four habitat levels: (1) breeding predominantly in coniferous forest, (2) breeding predominantly in mixed and deciduous forest, (3) breeding predominantly in farmland habitat, and (4) breeding predominantly in urban areas. In the last instance, just five species were classsified as breeding in urban areas (see Additional file 1: Table S2), and this was necessary because they had a predominantly urban distribution and therefore could not be identified as either forest or farmland breeders. Importantly, this approach does not involve circularity in the analyses of how breeding habitat influences urban occurrence frequency because these species did not have higher urban occurrence frequency than all the other groups (urban: mean 0.30, range 0.01-0.71, farmland species: mean 0.37, range 0.00-1.00, coniferous forest species: mean 0.04, range 0.00-0.34, mixed and deciduous forest species: mean 0.33, range 0.00-0.99; Mann–Whitney *U*-tests: urban vs. farmland: *P* = 0.94, urban vs. coniferous: *P* = 0.003, urban vs. mixed/deciduous: *P* = 0.98). Thus, being classified as an urban breeder did not by definition imply being common in the urban sites. Furthermore, an analysis excluding these five species returned qualitatively similar results (data not shown).

Finally, we examined the effects of nest site location as this variable has previously been shown to influence urban bird community composition (e.g. [5,8,19,20]) and because vegetation structure differs dramatically between urban sites and those in the surrounding environment. For example, ground based vegetation tends to be lacking in urban areas, instead it is often replaced with short grass, which is expected to influence the possibility of ground nesting birds to find suitable nesting sites. Nest site location was classified into four levels: ground, low in bushes (<2 m above ground), high in trees (>2 m above ground), and in cavities or other concealed sites. Information on nest sites were taken from the standard reference work on Norwegian birds [45]. Values for ecological variables used for each species are provided in Additional file 1: Table S2).

### Phylogeny

Species values may not represent independent data points for analysis due to similarities inherited through shared ancestry [54]. Therefore, we conducted all comparative analyses controlling for phylogeny (see below). We generated a phylogeny for the 90 species included in our main dataset from the recently published time-calibrated molecular phylogeny of all extant avian species [55]. More specifically, we downloaded 1000 phylogenetic trees for our species from those available at www.birdtree.org using the Hackett sequenced species backbone. Following Jetz et al. [55], we used the Hackett backbone for our analyses due to the more extensive genomic scope of loci used to construct this topology. We then summarised the sample of trees onto a single Maximum clade credibility (MCC) tree with median node heights using TreeAnnotator v1.7.4 (BEAST [56]). The phylogeny is shown in Additional file 4: Figure S2).

### Statistical analyses

To account for the non-independence of data points due to shared ancestry of species we used a generalized least-squares (GLS) approach in a phylogenetic framework (PGLS) to perform multiple regression analysis. The PGLS approach allows the estimation (via maximum likelihood) of a phylogenetic scaling parameter (λ), which indicates the degree of phylogenetic dependency in correlations among traits. Specifically, values of λ = 1 indicate complete phylogenetic dependence (i.e. traits covary in direct proportion to their shared evolutionary history), while values of λ = 0 indicate phylogenetic independence of trait covariance (i.e. trait coevolution is independent of phylogeny). Following the estimation of λ values, we used likelihood-ratio tests to compare the model where λ assumes its maximum likelihood value against models with values of λ = 0 or 1 [57].

For our main analysis, we included occurrence frequency (proportion of sites used) in urban sites as our response variable and occurrence frequency (proportion of sites used) in sites surrounding Oslo, relative brain mass, migratory status, breeding habitat and nest site location as independent variables in our model. As detailed above, we examined the effect of relative brain mass by including both (log-transformed) brain mass and body mass as predictor variables in our model. This model incorporated occupancy data from all 90 species based on data from the 93 urban sites and the 176 rural sites.

Next, we repeated this analysis using a restricted subset of urban sites. Specifically, we examined whether urban occurrence frequency was associated with occurrence frequency in surrounding sites, relative brain mass, migratory status, breeding habitat and nest site location using data from only 22 sites occurring within the inner city centre, which represent a highly urbanized environment. These sites were generally smaller than those in the total set of urban sites (i.e. median 2.5 ha, range 0.6 - 24.2 ha), and were heavily used by people.

For these models, however, collinearity between the predictor variables brain and body mass was problematic (i.e. variance inflation factors (VIF) = 10.6 and 11.5, body and brain mass respectively, exceeding the threshold of 10 [58,59]), as a result of the strong correlation between brain mass and body mass (*r* = 0.93 [95%CI = 0.90 – 0.94], *df* = 88, *t* = 22.83, *P* < 0.0001, λ = 0.99 ^<0.0001,0.73^). Consequently, following recommendations [60,61], we performed a sequential PGLS regression in which the effect of relative brain mass was assessed by including (log-transformed) brain mass as the original variable and body mass as the residuals from a PGLS regression of (log-transformed) body mass on brain mass (see also [42]). Though a statistically sound approach, the use of sequential regression does make the interpretation of the predictor variables less intuitive. Specifically, the effect of brain mass (i.e. the focal variable) is interpreted as the unique effect of brain mass in addition to the effect it already made through its relationship with body mass, while information on body mass (i.e. the residualized variable) *per se* is lost. Thus sequential regression is related to path analysis methods where variables can act both directly and indirectly through their relationships with other variables [61]. Because we also wanted to investigate the effect of body mass in our analyses, however, we repeated each sequential regression with body mass as the focal variable and brain mass included as the residuals from a PGLS log-log regression of brain mass on body mass. For these analyses, results for all predictor variables other than brain mass and body mass were identical. We therefore only report the results relevant to these two variables (i.e. body mass and residual brain mass) for these subsequent sequential regressions. Importantly, this approach eliminated collinearity among the predictors (all VIF < 2.2) and results reflected those obtained in the previous multiple regression models.

For these models, both continuous and categorical variables were included as predictor variables in our multiple regressions. Moreover, two of the three categorical variables used in our study (i.e. breeding habitat and nest site location) were comprised of four different levels. Consequently, because results relevant to the overall effect of each ecological variable cannot be obtained using the summary function for the PGLS (as this returns parameters for n-1 levels of each categorical factor using the remaining level as the reference level; i.e. “treatment contrasts”), we also summarized parameter effects from a multiple regressions (GLS) using a Type III (simultaneous) sum of squares (i.e. ANCOVA). This approach provided test statistics and the associated *P*-values for each of the six predictor variables (occurrence frequency in surrounding sites, brain mass, body mass, migratory status, breeding habitat and nest site location), and was appropriate in our case because the maximum likelihood estimate of λ was 0 (see results). Importantly, these results confirmed the earlier results from the PGLS. For simplicity, we present the Type III (simultaneous) sum of squares summaries in the main text and provide results of the full PGLS model in supplementary materials.

In addition to our main anlaysis, we also performed a PGLS to assess the reationship between occurence in urban sites and relative brain mass using a reduced model approach (i.e. when frequency in surrounding areas and ecological variables were not included in the model); this analysis was done to compare our results to those reported in previous studies. Note, however, that this simplified model had a higher Akaike’s information criterion (AIC) score compared to the full model of our main analysis (∆AIC = 108.8). Finally, in this instance, despite the correlation between brain mass and body mass, there was no serious collinearity among the predictor variables (i.e. VIF = 6.9).

For all PGLS models, we rejected the use of Bonferroni correction as it increases the probability of committing type II errors [62]. Instead, to assess the strength of the relationship between dependent and predictor variables, we calculated standardised effect sizes (partial *r*) from the *t*-values from multiple regressions [58]. We also calculated the noncentral 95% confidence intervals (CIs) for effect sizes from the *t*-values in our statistical models [58,59] confidence intervals excluding zero indicate statistical significance at level α = 0.05 [59].

Finally, in order to more fully explore the effects of the breeding habitat and nest site location on urban occurrence, we performed posthoc pairwise comparisons of all levels using a re-ordering approach. More specifically, for both breeding habitat and nest site location, we reran our main analysis model using PGLS, setting a different categorical level as the reference level each time. Thus the breeding habitat model was run 4 times, with a different level (i.e. urban, farmland, coniferous forest and mixed/deciduous forest) set as the reference level on each run. Similarly, the model for nest site location was run 4 times with one of the four levels (i.e. cavity, ground, low and high) set as the reference level on each run. In this instance, we calculated false discovery rates (FDR) for *P*-values to correct for multiple comparisons.

All variables were either normally distributed or ln-transformed to improve normality, and all occurrence frequency data were expressed as proportions and arcsine-square root transformed prior to analysis. Analyses were performed using R 3.0.2 [63] and the R packages ‘caper’ and ‘nlme’. Following Zuur et al. ([64], page 129) modeling assumptions (i.e. normality of residuals and homogeneity of variance) were validated through visual inspection of graphical model evaluation plots.

### Analyses of previously published data

We used data on densities of land bird species during the breeding season within and outside six cities (Livorno, Pisa, Madrid, Angers, Rennes, Heinola) in four European countries [5,65-67]. This represented, to our knowledge, all published studies with relevant types of data. None of these studies specifically presented correlation analyses of species densities within and outside urban areas, but they did examine the issue of how urbanization affected bird community composition. For each study, there were data from 2–3 urban zones: city centre, city suburbs and (for two cities only) other urban areas (e.g. parks), and species data were presented as densities (or proportions of total numbers observed) as opposed to the analyses from Oslo where we used occupancy data. We tested whether density outside cities was related to density in each of the urban zones, while also including the potential effect of relative brain mass (ln-transformed body mass and brain mass) of each species; data on brain mass were taken from the same sources as reported above, and we used corresponding body masses reported by these sources. Information on brain mass was missing for eight species, thus analyses had reduced sample size compared to original publications (seven less for Madrid, one less for Angers, Rennes and Heinola).

Phylogenies and comparative analyses were conducted as described above. Briefly, for each city we generated a species phylogeny by downloading 1000 phylogenetic trees for our species and summarising this sample of trees onto a single MCC tree. Next, for each of the six European cities we performed PGLS regressions with urban density as the dependent variable and both density outside urban areas and log-transformed brain mass and body mass as independent predictor variables, running seperate models for each urban zone. For four cities – Madrid, Angers, Rennes and Heinola – there was no serious collinearity among the predictor variables (all VIF < 8.7). However, for two cities – Livorno and Pisa – there was collinearity among predictor variables (i.e. VIF > 12). Thus we again used sequential regression to remove collinearity among predictors and we present the results of these analyses for these two cities. Moreover, to examine the effects of both brain mass and body mass we performed the sequential regression once with brain mass as the focal variable and once with body mass as the focal variable. When necessary, variables were transformed prior to analysis to meet modelling assumptions, and modelling assumptions were validated through visual inspection of model evaluation plots following Zuur et al. ([64], page 129). Finally, as before, we calculated effect size (*r*) to determine the strength of the relationship between traits of interest. We also calculated 95% noncentral confidence limits for each *r* in order to assess statistical significance: confidence intervals excluding zero indicate statistical significance at the level of α = 0.05.

## Results

### Urban birds in Oslo

Overall, species lists for urban and rural sites together included 90 species. Bird species recorded during the urban censuses (*N* = 60) occurred on average in 34.7 of the 93 urban sites in Oslo (range 1–93; 53 species occurred in > 1 site). Species recorded in the surroundings of Oslo (*N* = 89) occurred on average in 61.4 of the 176 sites (range 3–175).

**Figure 1:**
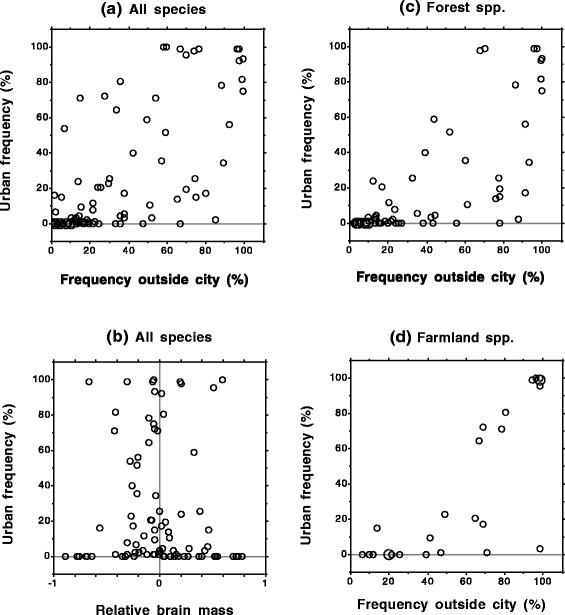
Relationships between rural and urban commonness, and brain mass and urban commonness, of bird species in Oslo. Frequency of occurrence of bird species in urban green spaces in Oslo compared to frequency in the surroundings of Oslo for **(a)** all species (*N* = 90), **(b)** compared to relative brain mass for all species (*N* = 90) measured as residuals from a regression of brain mass on body mass, **(c)** compared to frequency in the surroundings of Oslo for forest species (*N* = 62) , and **(d)** for farmland species (*N* = 26). Frequencies were percentage of sites where each species was recorded as present (number of sites in urban area: 93; number of sites outside city: 137 forest sites, 51 farmland sites, 176 sites in total). Unlike all analyses, figures do not control for phylogeny.

We found that frequency in the surrounding areas, habitat and nest site location significantly predicted the occurrence of species in urban sites (Table 1, see also Additional file 1: Table S3 and Figure 1a). More specifically, frequency in the surrounding areas strongly and positively predicted urban occurrence (partial *r* = 0.81). In contrast, migration was not a significant predictor of urban commonness (Table 1). There were also no significant effects of body mass or brain mass when brain mass was the focal variable (Table 1, see also Additional file 1: Table S3 and Figure 1b) or when body mass was the focal variable (body mass: *r* = 0.04 [95% CI = −0.18 – 0.25], df = 79, β = 0.007 ± 0.02, *t* = 0.34, *p* = 0.74; residual brain mass: *r* = −0.20 [95% CI = −0.39 – 0.02], df = 79, β = −0.17 ± 0.10, *t* = −1.77, *p* = 0.08, λ = 0.0 ^1.0, <0.0001^).

**Table 1:** **Factors affecting urban commonness of bird species in Oslo**

**Predictor**	***df***	***F***	***P***
Frequency in surrounding sites	1	152.61	**<0.0001**
Habitat type	3	18.50	**<0.0001**
Migration	1	1.98	0.16
Nest location	3	6.17	**0.0008**
Brain mass	1	0.17	0.68
Residual body mass	1	3.91	0.052

Results from ANCOVA (summarized with a sequential sum of squares) showing associations between the frequency of occurrence of bird species (*N* = 90) in urban sites (*N* = 93) in Oslo and six predictor variables (frequency of occurrence in surrounding sites, habitat type, migration, nest site and relative brain mass [i.e. ln-transformed brain mass and residual body mass]). Significant values (*P* < 0.05) are in bold. See Additional file 1: Table S3 for full results from summarization of PGLS model.

When urban sites were restricted to those sites within the more heavily urbanized inner city zone, frequency in the surrounding areas again strongly and positively influenced the likelihood of a species occurring in urban sites (partial *r* = 0.75). Habitat and nest site location also influenced the likelihood of a species occurring in urban sites, whereas migration was not associated with urban commonness (Table 2, see also Additional file 1: Table S4). Finally, our analyses showed that neither brain mass nor body mass were significant predictors of urban commonness. Specifically, when brain mass was investigated as the focal trait in the sequential regression, we found no signficant relationship between these variables (Table 2, see also Additional file 1: Table S4). Similarly, when body mass was the focal trait in the sequential regression, there was no association between urban commonness and body mass (body mass: *r* = 0.07 [95% CI = −0.15 – 0.28], df = 79, β = 0.001 ± 0.002, *t* = 0.62, *p* = 0.54; residual brain mass: *r* = −0.23 [95% CI = −0.42 – -0.01], df = 79, β = −0.02 ± 0.01, *t* = −2.10, *p* = 0.04, λ = 0.0 ^1.0, <0.0001^). Though this later sequential regression showed a significant association between residual brain mass and urban commonness, we do not interpret this as a significant effect of relative brain mass on urban commonness as the interpretation of the residualised variable in a sequential regression is difficult. Nonetheless, it is worth noting that this relationship was negative and thus opposite to findings in previous studies [13,14], i.e. birds with bigger brains were less common in urban sites.

**Table 2:** **Factors affecting urban commonness of bird species in Oslo, with subset of urban sites from the highly urbanized inner city centre only**

**Predictor**	***df***	***F***	***P***
Frequency in surrounding sites	1	101.78	**<0.0001**
Habitat type	3	19.71	**<0.0001**
Migration	1	1.56	0.22
Nest location	3	7.04	**0.0003**
Brain mass	1	0.10	0.75
Residual body mass	1	5.89	**0.018**

Results from ANCOVA (summarized with a sequential sum of squares) showing associations between the frequency of occurrence of bird species (*N* = 90) in urban sites (*N* = 22) in Oslo and six predictor variables (frequency of occurrence in surrounding sites, habitat type, migration, nest site and relative brain mass [i.e. ln-transformed brain mass and residual body mass]). Significant values (*P* < 0.05) are in bold. See Additional file 1: Table S4 for full results from summarization of PGLS model.

Importantly, urban living was also most strongly predicted by occurrence in the surrounding areas when the dataset was restricted to only forest species or only farmland species (see Additional file 1: Tables S5-S6, see also Figure 1c,d), and when we estimated rural commonness using abundance data (i.e. point count data and national population size; see Additional file 1: Tables S7-S8). Finally, our results were not affected by which source data for brain mass were used (see Additional file 1: Tables S9-S10).

We also found that relative brain mass did not explain frequency within the city when our model did not include frequency in the surroundings or ecological factors. Specifically, neither body mass (*r* = −0.008 [95% CI = −0-21 – 0.20], df = 87, β = −0.007 ± 0.09, *t* = −0.08, *p* = 0.94) or brain mass (*r* = −0.09 [95% CI = −0-28 – 0.12], df = 87, β = −0.111 ± 0.14, *t* = −0.81, *p* = 0.42, λ = 0 ^1.0, <0.0001^) were associated with our index of urbanization (i.e. frequency of occurrence in urban sites) in the reduced model.

**Figure 2:**
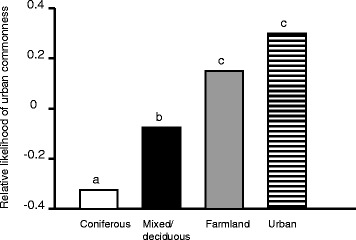
Relative likelihood of species occurring in urban sites as a function of breeding habitat. Bars marked with different letters **(a**, **b** **or** **c)** differ significantly from one another (posthoc pairwise comparisons, *P* < 0.05). The value shown for each habitat category is the estimated intercept (estimated frequency of urban occurrence) from posthoc comparisons when that category is set as the reference level (see main text for statistical details). The Y-axis indicates the relative likelihood, rather than absolute probability, of becoming urbanized.

**Figure 3:**
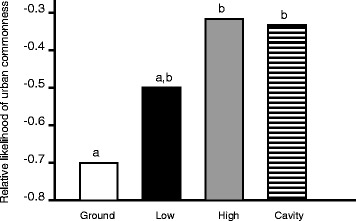
**Relative likelihood of species occurring in urban sites as a function of nest site location.** Bars marked with different letters (a or b) differ significantly from one another (posthoc pairwise comparisons, *P* < 0.05). The value shown for each nest site category is the estimated intercept (estimated frequency of urban occurrence) from posthoc comparisons when that category is set as the reference level (see main text for statistical details). The Y-axis indicates the relative likelihood, rather than absolute probability, of becoming urbanized.

While a species’ breeding habitat influenced its likelihood of occurring in urban spaces, the effects of different types of habitat were not equal. Specifically, species breeding in farmland areas were more likley to be found in urban centres in Oslo when compared to those breeding in forests. Moreover, in the comparison of the two forest types (i.e. coniferous vs. mixed/deciduous), species in coniferous forests were the least likely to be found in urban sites (Table 3, Figure 2). Similarly, the effect of nest site location differentially influenced the occurrence of species in urban sites. Specifically, cavity and high nesting species, which did not differ in their likelihood of occurring in urban sites, were significantly more likely to be found in urban sites compared to ground nesting species. In contrast, low nesting species did not differ from either ground nesters or the cavity/high nesting group in terms of occurrence in urban sites (Table 4, Figure 3).

**Table 3:** **Post-hoc pairwise comparisons of the four breeding habitat types and their effect on species occurence in urban sites**

	**Pairwise comparison**		
**Reference level**	**Urban**	**Farmland**	**Coniferous forest**
Farmland	β = 0.14 ± 0.13		
	*t* = 1.04, *P* = 0.30		
Coniferous forest	β = 0.64 ± 0.13	β = 0.50 ± 0.07	
	*t* = 4.76, *P* <0.0001	*t* = 6.84, *P* <0.0001	
Mixed/deciduous forest	β = 0.38 ± 0.14	β = 0.24 ± 0.07	β = −0.26 ± 0.07
	*t* = 2.80, *P* = 0.0077	*t* = 3.48, *P* = 0.0012	*t* = −3.56, *P* = 0.0012

*P*-values are adjusted for multiple comparison using false discovery rates (FDR).

**Table 4:** **Post-hoc pairwise comparisons of the four nesting site locations and their effect on species occurence in urban sites**

	**Pairwise comparison**		
**Reference level**	**Ground**	**Low**	**High**
Low	β = −0.20 ± 0.10		
	*t* = −1.99, *P* = 0.09		
High	β = −0.37 ± 0.09	β = −0.18 ± 0.09	
	*t* = −4.03, *P* = 0.0007	*t* = −1.89, *P* = 0.09	
Cavity	β = −0.36 ± 0.10	β = −0.16 ± 0.09	β = 0.01 ± 0.07
	*t* = −3.85, *P* = 0.0007	*t* = −1.80, *P* = 0.09	*t* = 0.16, *P* = 0.87

*P*-values are adjusted for multiple comparison using false discovery rates (FDR).

### Other European cities

In general, species density in the surroundings was the only significant predictor of urban density in six additional cities across Europe: Livorno, Pisa (Italy), Angers, Rennes (France), Madrid (Spain) and Heinola (Finland) (Table 5, see also Additional file 1: Tables S12-S17). Moreover, effect sizes of density in the surroundings were generally large (*r* = 0.12-0.96, median 0.81; Additional file 1: Tables S12-S17). The one exception to this pattern was the density of species in the city centre of Madrid, for which none of the predictors were significantly associated with urban density (Table 5, Additional file 1: Table S14). In contrast, brain mass was not significant in any of 14 comparisons, and effect sizes were generally low (*r* = −0.28-0.31, median −0.05; Additional file 1: Tables S12-S17). Similarly, we found no signficant effects of body mass on urban commonness (Table 5, see also Additional file 1: Tables S12-S17). This was also the case when analyses were performed using sequential regressions. Specifically, there were no significant associations between body mass and urban commonness in models examining Livorno city centre (body mass: *r* = 0.19 [95% CI = −0.29 – 0.57], df = 16, β = 0.02 ± 0.02, *t* = 0.78, *p* = 0.44; residual brain mass: *r* = 0.24 [95%CI = −0.25 – 0.60], df = 16, β = 0.12 ± 0.13, *t* = 0.99, *p* = 0.34, λ = 0.0 ^1.0, 0.0007^) or suburbs (body mass: *r* = 0.28 [95%CI = −0.21 – 0.62], df = 16, β = 0.013 ± 0.01, *t* = 1.15, *p* = 0.27; residual brain mass: *r* = −0.03 [95%CI = −0.46 – 0.42], df = 16, β = −0.007 ± 0.07, *t* = −0.10, *p* = 0.92, λ = 0.0 ^1.0, 0.0008^). Similarly, there were no significant associations between body mass and urban commonness in models examining Pisa city centre (body mass: *r* = 0.13 [95% CI = −0.34 – 0.53], df = 16, β = 0.01 ± 0.02, *t* = 0.52, *p* = 0.61; residual brain mass: *r* = −0.08 [95%CI = −0.49 – 0.38], df = 16, β = −0.04 ± 0.13, *t* = −0.31, *p* = 0.76, λ = 0.0 ^1.0, 0.001^) or suburbs (body mass: *r* = −0.21 [95%CI = −0.58 – 0.28], df = 16, β = −0.008 ± 0.01, *t* = −0.85, *p* = 0.41; residual brain mass: *r* = −0.11 [95%CI = −0.51 – 0.36], df = 16, β = −0.03 ± 0.06, *t* = −0.43, *p* = 0.67, λ = 0.0 ^1.0, 0.002^).

**Table 5:** **Factors affecting urban density of bird species in six European cities**

**City**	**Surroundings**	**Body mass**	**Brain mass**
Livorno, Italy (*N* = 20 species)			
City centre	**<0.0001**	0.50*	0.21
Suburbs	**<0.0001**	0.73*	0.24
Pisa, Italy (*N* = 20 species)			
City centre	**< 0.0001**	0.70*	0.67
Suburbs	**< 0.0001**	0.86*	0.26
Madrid, Spain (*N* = 46 species)			
City centre	0.42	0.30	0.28
Suburbs	**0.003**	0.60	0.51
Other urban areas	**0.016**	0.56	0.46
Angers, France (*N* = 43 species)			
City centre	**< 0.0001**	0.34	0.81
Suburbs	**< 0.0001**	0.27	0.22
Rennes, France (*N* = 33 species)			
City centre	**< 0.0001**	0.50	0.55
Suburbs	**< 0.0001**	0.79	0.83
Heinola, Finland (*N* = 48 species)			
City centre	**0.0005**	0.48	0.63
Suburbs	**< 0.0001**	0.54	0.72
Other urban areas	**< 0.0001**	0.35	0.21

Summary of results of analyses of interspecific associations (controlling for phylogeny) between species density in urban areas in six European cities and the predictor variables density outside the city and relative brain mass (i.e. ln-transformed body mass and brain mass; * indicates the use of residual body mass from sequential regression). For each city there were 2–3 different urban zones. The table shows *P*-values of predictor variables. Significant values (*P* < 0.05) are in bold. See Additional file 1: Tables S12-17 for full output of analyses.

## Discussion

### Relationship between commonness in rural and urban areas

This study showed that the composition of the urban bird community in Oslo reflected that of surrounding areas: species that were widespread in the surroundings were also more common within the city. This relationship was also present for both forest and farmland species independently. In addition, we found that species nesting on the ground were less likely to occur in urban environments relative to species breeding in either high or cavity nesting sites, and that species requiring coniferous forest breeding habitat were less common in urban sites compared to species breeding in the other habitat types. These results support the idea that urban bird community composition may be the result of immigration from surrounding landscapes [23], similar to a propagule pressure [26,27]. Our results are also consistent with two recent studies that showed that rural and urban commonness were correlated for several cities, including Oslo [20,31].

Our analyses of previously published data from six additional European cities also found a relationship between urban and rural bird assemblages. Moreover, in most cities the relationship between urban and rural occurrence was very strong, even in analyses using data from the most urbanized areas which largely lacked parks and other green spaces. The data set for Madrid [67] was strongly affected by ‘urban exploiters’ (e.g. *Columba livia*, *Passer domesticus*), species which were common in urban areas but more or less absent in rural areas, which may explain the weaker relationship between density outside and within the city. Importantly, despite these analyses using different types of data (i.e. relative densities versus presence/absence used for Oslo), similar results were expected because widespread species generally have higher densities [32,34,35]. Moreover, when abundance data were used as a measure of commonness outside Oslo, our results remained the same even though the source data were from wider areas and thus did not account for the local source populations as well as presence/absence data.

In conclusion, the relationship between rural and urban commonness was robust against which types of data were used (presence/absence or density), was consistent across a large geographical area (the whole of Europe) and had a consistently large effect size. Thus, our results suggest that community assemblage via random dispersal plays an important role in determining which species become urban, in combination with effects of habitat and nest site selection (see below). This is in line with the results from a recent study which also showed that the loss of species in urban areas is partly due to random processes and partly due to lack of appropriate adaptations for urban living [20], although this study concluded that appropriate adaptations were most important. Furthermore, our findings in combination with results from previous studies [20,31] suggest community assemblage via random dispersal is a general process spanning a broad geographic range.

### Degree of urbanization

In this study, urban sites within Oslo city consisted of parks, cemeteries and other green spaces. One could argue that the species we recorded included many which are not truly urbanized, but simply utilize patches of natural vegetation within the city. For some species which were present mostly in the suburbs this may have been true, but the results of our analyses remained the same when restricted to urban sites in the heavily urbanized inner city centre where the number of species present was much lower (only 28 species present in two or more sites) and where only one of the 22 sites had any natural vegetation. Theoretically, one might restrict studies of urbanization to only those sites which lack vegetation entirely in order to focus on species able to adapt to the most heavily modified areas. However, in most cities such areas are used by only a few species, and it has been commented that only a small proportion of the bird species that are found in cities are well adapted to urban environments [18]. In Oslo, the most urbanized areas which lack trees and other vegetation are used by only a handful of land bird species (mainly *Columba livia*, *Apus apus*, *Corvus corone*, *Motacilla alba* and *Passer domesticus*, S. Dale, personal observations) and we do not think it is useful to restrict analyses aimed at understanding the process of urbanization to such a limited number of species. Moreover, most previous studies have taken a similar approach as ours, focusing on the full range of species utilizing urban areas (e.g. [5,8,13,14,18,67]), thus also allowing our results to be comparable to those in the relevant literature.

### Effects of ecological variables

As expected, species nesting in safer sites or higher above ground were relatively more common in urban areas, in line with findings from other studies (e.g. [8,19]). Furthermore, we found that breeding habitat selection influenced the likelihood of species occurring in urban sites. The most important finding was that farmland species were more often present in urban sites than species breeding in mixed or deciduous forest. Here it should be noted that several common species classified as belonging to farmland habitat use farm buildings and yards extensively (such as *Pica pica*, *Corvus corone*, *Sturnus vulgaris*, *Passer montanus*, *Motacilla alba*), which possibly make them preadapted to exploiting urban areas. The finding that species from coniferous forest habitat were less likely to become urban was expected because the urban areas in Oslo generally lacked this habitat, but this result underlines that availability of suitable habitat is a prerequisite for urban invasion. Finally, although not important for our main conclusions, we note that the apparent pattern in Figure 2 that species breeding in urban habitats were most likely to become urban should be interpreted with reference to how we classified urban species: they were species with predominantly urban distribution so that their occurrence frequency in rural areas was by definition low. Thus, the analyses simply showed that these species were more common in urban sites than their rural commonness would predict. Note, however, that the urban species still did not have higher mean occupancy rates than farmland and mixed/deciduous forest species (cf. Methods and Additional file 1: Table S2).

### Effects of relative brain mass and body mass

Our analyses showed that body mass-corrected brain mass was not significantly associated with species occurrence in urban areas, even in an analysis in which frequency in surroundings was not included. We failed to find significant effects of relative brain mass in independent analyses from seven European cities. In the comprehensive analysis of data from Oslo, the estimated slope of brain mass was negative (but not significant), opposite to the prediction from the brain mass hypothesis. Even the upper confidence limits of the effect sizes of relative brain mass, although positive, were in general small, suggesting that increasing the power of the analyses would still lead to the conclusion that brain mass has a weak effect on urban commonness compared to rural commonness and ecological variables.

Our finding that relative brain mass was not significantly related to urban commonness is consistent with a number of recent studies [18-20]. However, it stands in contrast to other studies indicating that adaptation to urban life is related to brain size [13,14]. We suggest that if brain size is relatively larger among urban birds, this may be mediated through an effect of brain size on general commonness of species (e.g. that generalists have relatively larger brains and generalists are usually common), but commonness in source areas is what affects urban commonness *per se*. Ducatez et al. found that dietary generalism, and not habitat generalism, is correlated with relative brain size [68] suggesting that a relationship between brain size and urban invasion could be mediated by a broad diet. On the other hand, to our knowledge there has been no specific analyses of the relationship between brain size and rural abundance, though at least one study has examined the relationship between population decline and relative brain size [69].

Interestingly, we also found that among lineages well known for their large brains (e.g. Corvidae and Paridae) several species in our sample were actually rare or absent from the urban areas in Oslo despite being widespread in the surroundings (*Garrulus glandarius*, *Nucifraga caryocatactes*, *Corvus corax*, *Lophophanes cristatus*, *Poecile montanus*). This contrasts with an earlier study which indicated that a larger proportion of species in large-brained families were recorded in cities [14]. More specifically, that study found that, among 82 passerine bird species occurring around 12 cities in France and Switzerland, species with larger brains were more likely to be classified as successful urban colonizers (i.e. recorded in the city centre of at least one of the 12 cities) [14]. However, their analyses ignored ecological variables and the potential effect of rural abundance. Based on data on presence/absence of species in cities reported in their electronic appendix, we note that species classified as occurring in one or more cities had larger national population sizes (median 1,375,000, *N* = 38, using population data from France [70] because 11/12 cities were in France) relative to species which did not occur in cities (median = 45,000, *N* = 44; *U*-test: *z* = −6.49, *P* < 0.0001). Thus, these data may also be compatible with the idea that rural abundance may affect urban occurrence.

Carrete and Tella [13] also found that urban invasiveness was related to relative brain size among 27 bird species in Bahía Blanca, Argentina, and suggested that the effect of brain size was mediated through an effect of brain size on flight initiation distances. However, they also found that urban invasiveness was related to rural abundance, but they did not perform multivariate analyses to assess the relative effect of brain size versus rural abundance on urban abundance. Thus, pending more detailed analyses, these findings may also be compatible with the idea that relative brain size may affect rural abundance, and, subsequently, rural abundance determines urban abundance.

In contrast, Kark and coworkers [18] did not find any significant changes in mean relative brain size of species along an urbanization gradient in Jerusalem, but suggested that ability to exploit urban areas depended on a combination of life history traits such as diet, sociality, migration and nest sites. Similarly, Evans and coworkers [19] analysed densities of 55 species in about 3000 British urban and rural sites, but found no evidence that relative brain size influenced responses of species to urbanization. Instead, the authors concluded that generalist species were most favoured by urban development. Finally, Sol and coworkers found no effect of brain size when comparing 358 species classified as urban exploiters or urban avoiders from 22 locations from five continents [20].

There is a vast literature suggesting that brain size may have profound ecological implications (see e.g. review in [21]). From this, it is an obvious possibility that brain size may play a role in adaptation to urban life considering that this may require shifts in anti-predator behaviour [13], foraging behaviour [11], and nest site selection [17]. However, based on our review of previous studies on the relationship between brain size and urban life among birds, we find the evidence in favour of an effect of brain size to be equivocal, and our data clearly do not support such a hypothesis. However, we acknowledge that discrepancies between studies regarding the effect of brain size on urban living could be due to geographical variation in the importance of this trait [20].

Finally, our data provided some insight into the importance of body mass for urban living. Large-bodied species might cope with urban environments better e.g. because of larger fat deposits which may buffer agains variable food supply, but on the other hand larger species have longer flight initiation distances [71] making them more susceptible to human disturbance, and larger species generally have larger area requirements which may limit their distribution in cities. Our results showed in general no significant effects of body mass, although in a few cases there were trends that larger species were more common in urban areas (Tables 1, 2 and Additional file 1: Table S9).

### Surrounding landscapes as sources for urban populations

Evans and coworkers [23] suggested that high population density in original habitat could promote invasion of urban areas. Our results provide strong support for this hypothesis. However, one important question is whether this means that urban areas are sinks relative to source populations outside cities. We think the answer may depend on the species in question. Urban establishment has been documented in detail in a few species; these studies show a pattern of multiple independent colonizations (*Columba palumbus* [72]; *Turdus merula* [23,73]), suggesting that urban populations are no longer dependent on immigration from surroundings. We believe this may be the case for several other widespread and common urban bird species. However, the urban bird community also contains a number of less common species, with much more variable occurrence. Unfortunately, there are no relevant studies providing information on whether these species have self-sustaining urban populations or depend on a continuous supply of immigrants from surrounding areas (but see [74] for a review of differences in reproductive parameters of urban and rural populations). We suggest that the latter may be the case, and that studies aimed at determining the origin of individuals of such species are needed.

The strong quantitative relationship between commonness of rural and urban populations found in the present study suggests that regional spatial variation in source populations may be an important explanation for variation in bird communities between cities. Species which can occur in urban areas may have substantial regional variation in population density in rural areas and, hence, one may expect that spatial variation in species’ densities can cause adoption of urban life in some areas, but not others. We are not aware of any studies which have tested this idea. On the other hand, urbanization is a recent process, and there may also be time lags in adaptation to urban environments which may add further spatial variation in use of urban areas by species present in the regional species pool. This may explain why some species did not occur within Oslo despite being common in the surroundings and common in urban areas of other parts of Europe (e.g. *Turdus philomelos*, *Garrulus glandarius*). We predict that, over time, colonization pressure may cause such species to also become urban in Oslo.

## Conclusions

Our results showed that urban commonness was primarily determined by how common species were in rural areas in combination with habitat preferences and nest site locations, and not by the relative brain mass or body mass of species. Thus, rural bird populations play an important role in shaping urban bird communities. The number of bird species which have become fully adapted to urban life is low [18], and this suggests that high urban bird diversity may depend on (1) source areas outside cities with high diversity, and (2) limitations on urban sprawl to take into account that immigration rates may be distance dependent [75]. Thus, green spaces in urbanized areas may be compared to islands, and basic biogeographical principles relating immigration rates from source (mainland) areas may determine species richness [24,25]. Together with well known effects of park size and vegetation structure [76,77], the present findings may help city planners and wildlife managers to better understand how urban bird diversity can be maintained or increased.

### Availability of supporting data

The data set supporting the results of this article is available in the Data Dryad repository, doi: 10.5061/dryad.pq6d7 [78].

## Additional files

Additional file 1: Tables S1-S17. List of urban study sites in Oslo, life history and ecological variables of species included in analyses of the urban bird community in Oslo, additional analyses of data from Oslo, overview of previously published data from other European cities, and additional analyses of data from other European cities. Additional file 2: Figure S1. Map of urban study sites in Oslo. Additional file 3:
**Supplementary methods.** Evaluation of possible biases in data. Additional file 4: Figure S2. Evolutionary relationships of the 90 species from Oslo.
