# Supplementary material for: Commonness and ecology, but not bigger brains, predict urban living in birds
Source: BMC Ecol. 2015 Apr 11;15:12. doi: 10.1186/s12898-015-0044-x (PMC4412207; doi:10.1186/s12898-015-0044-x)
Supplement: Additional file 1: Tables S1-S17. — List of urban study sites in Oslo, life history and ecological variables of species included in analyses of the urban bird community in Oslo, additional analyses of data from Oslo, overview of previously published data from other European cities, and additional analyses of data from other European cities. [file 12898_2015_44_MOESM1_ESM.docx]

Additional file 1

Tables S1-S17

Commonness and ecology, but not bigger brains, predict urban living in birds

Svein Dale, Jan T. Lifjeld and Melissah Rowe

Table S1. List of urban study sites in Oslo.

________________________________________________________________________________________________________________________

Inner city centre sites (*N* = 22).

________________________________________________________________________________________________________________________

Frøyas have Skarpsnoparken Framneshaven

Hydroparken Akershus festning Slottsparken

Uranienborgparken Langårdsløkken Stensparken

St. Hanshaugen Vår Frelsers Gravlund Margaretakyrkan

Gamle Aker kirke Iladalen Birkelunden

Olaf Ryes plass Sofienbergparken Tøyenparken

Grønlands park Minneparken, Clemens park Gamlebyen Gravlund

Middelalderparken

________________________________________________________________________________________________________________________

Other urban sites (*N* = 71).

________________________________________________________________________________________________________________________

Vækerøparken - Sollerudstranda Ullern kirke Hoffsdammen

Skøien hovedgård Vestre Gravlund Frognerparken

Tørtberg - Marienlyst Vestre Aker kirke Nordre Gravlund

Berg Bakkehaug kirke - Lunden Voldsløkka

Bjølsenparken Torshovparken Torshov kirke

Torshovdalen Ola Narr - Tøyenbadet Hasle kirke

Kampen park Vålerengen kirke Valle-Hovin

Østre Gravlund Svartdalen Mærradalen

Husebyskogen Makrellbekken; Njårdhallen - Ring 3 Hovseter

Voksen kirke - Voksen gård Frantsebråten Makrellbekken; Holmen

Holmendammen Holmenbekken Ris kirke

Gaustad sykehus Gaustad (Risbekken - Sognsvannbekken) Havnajordet

Engebråten Grefsen kirke Muselunden

Bekkedalen Økernparken Bjerkebanen

Linderud gård Bredtvet kirke/fengsel Nordtvet besøksgård

Alna; Huken - Ammerud - Grorud Grorud kirke Hølaløkka

Romsåsfoten Rommensletta Tokerudbekken

Stovner kirke Østre Aker kirke Ulvensplitten

Alna; Smalvollveien Alna; Terminalveien Alfaset Gravlund - Arveset gård

Stubberudmyra Alnaparken Lindeberg besøksgård

Furuset kirke Fossumbekken Høybråten stasjon

Høybråten kirke Ellingsrudkollen Ekeberg

Brannfjell Nordre Skøyen hovedgård Godlia

Bergkrystallen Nordstrand kirke

________________________________________________________________________________________________________________________

Table S2. Life history and ecological variables of species included in analyses of the urban bird community in **Oslo**, together with proportion of sites within the city (*N* = 93) and outside the city (*N* = 176) in which they were recorded. See Methods for definitions of variables.

________________________________________________________________________________________________________________________

Species Habitat**^§^** Nest site**^¶^** Migration Body mass Brain mass^‡^ % of urban sites % of rural sites

(g) (g)

________________________________________________________________________________________________________________________

*Coturnix coturnix* Farmland Ground Migratory 89 0.80 0 2.84

*Phasianus colchicus* Farmland Ground Resident 1244 3.68 0 7.95

*Tetrastes bonasia* Coniferous Ground Resident 372 2.00 0 10.23

*Tetrao urogallus* Coniferous Ground Resident 3108 5.46 0 3.98

*Lyrurus tetrix* Coniferous Ground Resident 1112 3.70 0 19.32

*Apus apus* Farmland Cavity Migratory 38 0.70 70.97 53.98

*Streptopelia decaocto* Urban High Resident 188 1.52 16.13 1.70

*Columba livia* Urban Cavity Resident 270 2.49 53.76 6.82

*Columba oenas* Mixed Cavity Migratory 275 2.20 1.08 13.07

*Columba palumbus* Farmland High Migratory 495 2.38 98.92 96.02

*Crex crex* Farmland Ground Migratory 160 1.29 0 3.41

*Cuculus canorus* Coniferous Ground Migratory 111 1.46 1.08 19.32

*Scolopax rusticola* Mixed Ground Migratory 313 2.51 0 16.48

*Vanellus vanellus* Farmland Ground Migratory 227 2.16 0 8.52

*Pernis apivorus* Mixed High Migratory 830 7.20 0 4.55

*Buteo buteo* Coniferous High Migratory 921 7.90 0 3.98

*Accipiter gentilis* Coniferous High Resident 1100 7.72 0 4.55

*Accipiter nisus* Mixed High Resident 204 2.88 4.30 14.20

*Asio otus* Farmland High Migratory 276 5.61 0 2.27

*Strix aluco* Mixed Cavity Resident 472 9.08 0 10.80

*Aegolius funereus* Coniferous Cavity Resident 134 3.80 0 7.39

*Glaucidium passerinum* Coniferous Cavity Resident 66 2.50 0 6.25

*Jynx torquilla* Mixed Cavity Migratory 38 0.85 2.15 11.93

*Picoides tridactylus* Coniferous Cavity Resident 66 3.20 0 7.39

*Dendrocopos major* Mixed Cavity Resident 87 2.51 25.81 74.43

*Dendrocopos minor* Mixed Cavity Resident 22 1.20 3.23 10.23

*Dryocopus martius* Coniferous Cavity Resident 339 7.70 0 24.43

*Picus canus* Coniferous Cavity Resident 125 3.60 0 2.27

*Picus viridis* Mixed Cavity Resident 199 4.35 5.38 37.50

*Lanius collurio* Farmland Low Migratory 31 0.99 0 10.23

*Garrulus glandarius* Coniferous High Resident 162 4.15 0 32.95

*Pica pica* Farmland High Resident 223 5.34 100.00 57.95

*Nucifraga caryocatactes* Coniferous High Resident 193 5.71 0 21.59

*Corvus monedula* Farmland Cavity Resident 223 4.69 15.05 5.11

*Corvus corax* Coniferous High Resident 1185 15.31 0 17.05

*Corvus corone* Farmland High Resident 543 8.14 95.70 69.89

*Cyanistes caeruleus* Mixed Cavity Resident 11 0.65 97.85 73.86

*Parus major* Mixed Cavity Resident 18 0.85 98.92 97.16

*Lophophanes cristatus* Coniferous Cavity Resident 11 0.70 4.30 35.80

*Periparus ater* Coniferous Cavity Resident 9 0.51 13.98 65.34

*Poecile montanus* Coniferous Cavity Resident 11 0.79 0 47.16

*Poecile palustris* Mixed Cavity Resident 12 0.58 0 5.11

*Alauda arvensis* Farmland Ground Migratory 39 0.97 1.08 22.16

*Acrocephalus palustris* Farmland Low Migratory 12 0.39 0 5.68

*Hippolais icterina* Mixed Low Migratory 13 0.54 20.43 25.57

*Sylvia atricapilla* Mixed Low Migratory 20 0.67 78.49 88.07

*Sylvia borin* Mixed Low Migratory 21 0.62 51.61 59.09

*Sylvia curruca* Coniferous Low Migratory 11 0.53 25.81 30.11

*Sylvia communis* Farmland Low Migratory 14 0.56 20.43 23.86

*Delichon urbica* Farmland Cavity Migratory 16 0.50 22.58 29.55

*Hirundo rustica* Farmland Cavity Migratory 20 0.58 17.20 37.50

*Aegithalos caudatus* Mixed High Resident 8 0.50 3.23 12.50

*Phylloscopus sibilatrix* Mixed Ground Migratory 10 0.37 7.53 21.59

*Phylloscopus collybita* Mixed Low Migratory 8 0.38 11.83 21.59

*Phylloscopus trochilus* Mixed Ground Migratory 9 0.31 81.72 98.86

*Regulus regulus* Coniferous High Resident 6 0.38 17.20 80.11

*Sitta europaea* Mixed Cavity Resident 23 1.11 59.14 49.43

*Troglodytes troglodytes* Mixed Ground Migratory 9 0.50 19.35 69.89

*Certhia familiaris* Mixed Cavity Resident 10 0.55 10.75 51.14

*Sturnus vulgaris* Farmland Cavity Migratory 80 1.70 80.65 35.80

*Turdus viscivorus* Coniferous High Migratory 113 2.21 0 13.07

*Turdus philomelos* Coniferous Low Migratory 75 1.59 2.15 85.23

*Turdus iliacus* Mixed Low Migratory 68 1.22 55.91 92.05

*Turdus merula* Mixed Low Migratory 102 1.92 92.47 97.16

*Turdus pilaris* Farmland High Migratory 102 1.76 98.92 76.14

*Muscicapa striata* Mixed Low Migratory 16 0.53 35.48 56.82

*Erithacus rubecula* Mixed Ground Migratory 18 0.66 75.27 99.43

*Ficedula hypoleuca* Mixed Cavity Migratory 13 0.45 39.78 42.05

*Phoenicurus phoenicurus* Coniferous Cavity Migratory 16 0.54 2.15 18.18

*Oenanthe oenanthe* Urban Cavity Migratory 24 0.66 6.45 2.27

*Saxicola rubetra* Farmland Ground Migratory 18 0.67 1.08 14.77

*Prunella modularis* Mixed Low Migratory 20 0.71 15.05 75.00

*Passer domesticus* Urban Cavity Resident 30 0.92 70.97 14.77

*Passer montanus* Farmland Cavity Resident 24 0.79 72.04 27.27

*Anthus trivialis* Coniferous Ground Migratory 22 0.62 0 66.48

*Motacilla alba* Farmland Cavity Migratory 22 0.58 98.92 66.48

*Emberiza citrinella* Farmland Ground Resident 31 0.82 3.23 51.70

*Fringilla coelebs* Mixed High Migratory 23 0.77 93.55 99.43

*Fringilla montifringilla* Coniferous High Migratory 24 0.78 1.08 4.55

*Coccothraustes coccothraustes* Mixed High Resident 55 1.63 23.66 13.64

*Carpodacus erythrinus* Farmland Low Migratory 22 0.99 0 12.50

*Pyrrhula pyrrhula* Coniferous High Resident 32 0.97 3.23 37.50

*Chloris chloris* Farmland High Resident 30 0.89 100.00 59.66

*Carduelis cannabina* Farmland Low Migratory 18 0.67 9.68 15.34

*Carduelis flammea* Mixed High Migratory 14 0.60 1.08 16.48

*Loxia curvirostra* Coniferous High Resident 41 1.47 0 35.80

*Loxia pytyopsittacus* Coniferous High Resident 50 1.82 0 1.70

*Carduelis carduelis* Farmland High Resident 16 0.59 64.52 33.52

*Carduelis spinus* Coniferous High Migratory 13 0.56 34.41 89.20

*Serinus serinus* Urban High Migratory 12 0.65 1.08 0

________________________________________________________________________________________________________________________

**§** A few species were included in analyses of both forest and farmland species (*Columba palumbus*, *Turdus pilaris*, *Lanius collurio*), but because they had somewhat higher frequencies in farmland than in forest they were classified as farmland species in analyses comprising all species. The urban habitat category comprised species with predominantly urban occurrence in the study area (*Columba livia*, *Streptopelia decaocto*, *Passer domesticus,* *Oenanthe oenanthe* which occurs almost exclusively in disturbed sites in cities and in coastal areas in this part of Norway, and *Serinus serinus* which is rare in Norway and only occurs in urban areas).

**¶** *Cuculus canorus* was classified as a ground nester because the most common likely hosts in the study area nest on the ground (Haftorn 1971).

‡ The most recent publications were given highest priority; except that data for *Corvus corone* and *Sturnus vulgaris* were not taken from Sol et al. (2010) because body mass differed strongly from Norwegian data, and that Møller et al. (2005) was used as data source for *Cuculus canorus*.

Haftorn, S. (1971) *Norges Fugler*. Universitetsforlaget, Oslo, Norway.

Møller, A.P., Erritzøe, J. & Garamszegi, L.Z. (2005) Covariation between brain size and immunity in birds: implications for brain size evolution. *Journal of Evolutionary Biology*, **18**, 223-237.

Sol, D., Garcia, N., Iwaniuk, A., Davis, K., Meade, A., Boyle, W.A. & Székely, T. (2010) Evolutionary divergence in brain size between migratory and resident birds. *PLoS ONE*, **5**, e9617.Table S3. **Results of full PGLS models examining factors affecting urban commonness of bird species in Oslo.** Interspecific associations (controlling for phylogeny) between the frequency of occurrence of bird species (full sample, *N* = 90) in urban sites in Oslo and six predictor variables (frequency of occurrence in surrounding sites, habitat type, migration, nest site, (ln-transformed) brain mass and residual body mass). The model including the maximum-likelihood value of λ was compared against models including λ = 0 and 1; superscripts following λ estimates indicate p-values of the likelihood-ratio tests (first position: against λ = 0; second position: against λ = 1). Effect sizes (partial *r*) and their noncentral 95% confidence intervals (LCL, lower confidence limit; UCL, upper confidence limit) were calculated for each urban occurrence-predictor variable pair. Significant relationships are shown in bold.

| **Predictor** | **Estimate** ± **SE** | ***t_79_*** | ***P*** | **Partial *r*** | **LCL-UCL** | **λ** |
| --- | --- | --- | --- | --- | --- | --- |
| Frequency in surrounding sites | **0.997 ± 0.08** | **12.35** | **< 0.0001** | **0.81** | **0.73 – 0.86** | 0 ^1, <0.0001^ |
| Habitat – mixed/deciduous | **0.255 ± 0.07** | **3.56** | **0.0006** | **0.37** | **0.17 – 0.53** |  |
| Habitat – farmland | **0.500 ± 0.07** | **6.84** | **< 0.0001** | **0.61** | **0.46 – 0.71** |  |
| Habitat – urban | **0.637 ± 0.13** | **4.76** | **< 0.0001** | **0.47** | **0.29 – 0.61** |  |
| Migration | 0.096 ± 0.07 | 1.41 | 0.163 | 0.16 | -0.06 – 0.36 |  |
| Nest – ground | **-0.364 ± 0.10** | **-3.85** | **0.0002** | **-0.40** | **-0.55 – -0.20** |  |
| Nest – high | 0.011 ± 0.07 | 0.16 | 0.871 | 0.02 | -0.20 – 0.23 |  |
| Nest – low | -0.163 ± 0.09 | -1.80 | 0.076 | -0.20 | -0.39 – 0.02 |  |
| Brain mass | -0.015 ± 0.04 | -0.41 | 0.68 | -0.05 | -0.26 – 0.17 |  |
| Residual body mass | 0.115 ± 0.06 | 1.98 | 0.052 | 0.22 | -0.001 – 0.41 |  |

Table S4. **Results of full PGLS models examining factors affecting urban commonness of bird species in inner city sites in Oslo.** Interspecific associations (controlling for phylogeny) between the frequency of occurrence of bird species (full sample, *N* = 90) in urban sites (inner city sites only) in Oslo and six predictor variables (frequency of occurrence in surrounding sites, habitat type, migration, nest site, (ln-transformed) brain mass and residual body mass). The model including the maximum-likelihood value of λ was compared against models including λ = 0 and 1; superscripts following λ estimates indicate p-values of the likelihood-ratio tests (first position: against λ = 0; second position: against λ = 1). Effect sizes (partial *r*) and their noncentral 95% confidence intervals (LCL, lower confidence limit; UCL, upper confidence limit) were calculated for each urban occurrence-predictor variable pair. Significant relationships are shown in bold.

| **Predictor** | **Estimate** ± **SE** | ***t_79_*** | ***P*** | **Partial *r*** | **LCL-UCL** | **λ** |
| --- | --- | --- | --- | --- | --- | --- |
| Frequency in surrounding sites | **0.083 ± 0.008** | **10.09** | **< 0.0001** | **0.75** | **0.65 – 0.82** | 0 ^1, <0.0001^ |
| Habitat – mixed/deciduous | **0.026 ± 0.007** | **3.58** | **0.0006** | **0.37** | **0.17 – 0.53** |  |
| Habitat – farmland | **0.046 ± 0.008** | **6.22** | **< 0.0001** | **0.57** | **0.41 – 0.69** |  |
| Habitat – urban | **0.084 ± 0.014** | **6.11** | **< 0.0001** | **0.57** | **0.40 – 0.68** |  |
| Migration | 0.009 ± 0.007 | 1.25 | 0.22 | 0.14 | -0.08 – 0.34 |  |
| Nest – ground | **-0.042 ± 0.010** | **-4.29** | **< 0.0001** | **-0.44** | **-0.58 – -0.24** |  |
| Nest – high | -0.002 ± 0.007 | -0.21 | 0.83 | -0.02 | -0.24 – 0.19 |  |
| Nest – low | -0.009 ± 0.009 | -0.96 | 0.34 | -0.11 | -0.31 – 0.11 |  |
| Brain mass | -0.001 ± 0.004 | -0.32 | 0.75 | -0.04 | -0.25 – 0.18 |  |
| Residual body mass | **0.014 ± 0.006** | **2.43** | **0.02** | **0.26** | **0.05 – 0.45** |  |

**Additional models**

We performed several additional PGLS regression in order to explore how our results may be effected by a range of conditions. First, we examined the effects of all six predictor variables (frequency of occurrence in surrounding sites, habitat type, migration, nest site, (ln-transformed) brain mass and body mass) on the likelihood of a species occurring in urban sites when data were restricted to (1) only forest species (*N* = 62) and (2) only farmland species (*N* = 26) in order to check that results were not affected by potential habitat influences on species detectability. Additionally, to check if the use of presence/absence data for source areas gave similar results to the use of abundance data, we also performed a PGLS where source data for forest birds were taken from point counts (Haavik & Dale 2012). While these data were from a wider area (the whole of Oslo and Akershus counties) than that used in our analyses, the comparison is relevant because the same forest type (i.e. boreal forest) dominates throughout the area. Of the 43 species recorded in their study, we excluded four predominantly farmland species that occurred only at relatively low density in point counts (*Corvus corone*, *Saxicola rubetra*, *Chloris chloris*, *Emberiza citrinella*). We also performed a PGLS using Norwegian national population size estimates as source area data for all 90 species (BirdLife International 2004). Finally, because some have criticized the use of multiple sources for brain mass (Healy & Rowe 2007), we repeated the main analysis using brain data from the major single source (Mlikovsky 1989a,b, 1990) and a more recent single source (Møller et al. 2005).

*Results*

We found that frequency in surrounding sites remained the strongest predictor of urban commonness when analyses were restricted to either forest species only (Table S5) or farmland species only (Table S6). For forest species only, forest type (i.e. coniferous vs mixed/deciduous) was also a significant predictor of urban commonness (Table S5), whereas for farmland species nest site location also significantly influenced species occurrence within urban sites (Table S6). In contrast, neither brain mass or body mass were significantly associated with urban commonness (Table S5 – S6; sequential regression with body mass as focal variable: body mass: *r* = -0.06 [95%CI = -0.32 – 0.20], df = 53, β = -0.01 ± 0.03, *t* = -0.47, *p* = 0.64; residual brain mass: *r* = -0.22 [95%CI = -0.44 – 0.05], df = 53, β = -0.23 ± 0.14, *t* = -1.60, *p* = 0.12, λ = 0.0 ^1.0, <0.0001^).

Next, when species occurrence in the surrounding areas was based on abundance data (i.e. point counts and national population size), results mirrored those using presence/absence data. More specifically, for the model utilising point count data, both species abundance in the surrounding areas and habitat influenced the frequency of species in urban sites within Oslo, whereas neither nest site location or migration were significantly associated with urban commonness (Table S7). Similarly, neither body mass or brain mass were significantly associated with species frequency in urban sites (Table S7; sequential regression with body mass as focal variable: body mass: *r* = 0.09 [95%CI = -0.27 – 0.41], df = 29, β = 0.03 ± 0.06, *t* = 0.48, *p* = 0.63; residual brain mass: *r* = 0.02 [95%CI = -0.33 – 0.36], df = 29, β = 0.02 ± 0.27, *t* = 0.08, *p* = 0.93, λ = 0.97 ^0.04, 0.84^); NB. for this model we were unable to summarize the parameters using a Type III sum of squares due to the high lambda value (**λ** = 0.97). Similarly, when abundance was estimated via Norwegian national population size, species abundance, habitat and nest site location were all significantly associated with urban commonness, whereas migration was not (Table S8). In these models, body mass and brain mass were also not significantly associated with urban commonness (Table S8; sequential regression with body mass as focal variable: body mass: *r* = -0.11 [95%CI = -0.31 – 0.12], df = 79, β = -0.03 ± 0.03, *t* = -0.94, *p* = 0.35; residual brain mass: *r* = -0.19 [95%CI = -0.39 – 0.03], df = 79, β = -0.26 ± 0.15, *t* = -1.74, *p* = 0.09, λ = 0.0 ^1.0, <0.0001^).

Finally, our results were not affected by which source data for brain mass were used (Table S9 –S10), and in these models neither body mass or brain mass were significant predictors of urban occurrence. This was the case for the single source brain data (i.e. Mlikovsky 1989a,b, 1990) model: (Table S9; sequential regression with body mass as focal variable: body mass: *r* = 0.08 [95%CI = -0.15 – 0.30], df = 71, β = 0.02 ± 0.02, *t* = 0.70, *p* = 0.48; residual brain mass: *r* = -0.19 [95%CI = -0.40 – 0.04], df = 71, β = -0.16 ± 0.10, *t* = -1.66, *p* = 0.10, λ = 0.0 ^1.0, <0.0001^). This was also true for the more recent single source (Møller et al. 2005) model: (Table S10; sequential regression with body mass as focal variable: body mass: *r* = -0.02 [95%CI = -0.29 – 0.26], df = 48, β = -0.004 ± 0.03, *t* = -0.12, *p* = 0.91; residual brain mass: *r* = -0.13 [95%CI = -0.38 – 0.15], df = 48, β = -0.14 ± 0.15, *t* = -0.92, *p* = 0.36, λ = 0.0 ^1.0, <0.0001^).

BirdLife International (2004) *Birds in Europe, population estimates, trends and conservation status*. Cambridge, BirdLife International.

Haavik, A. & Dale, S. (2012) Are reserves enough? Value of protected areas for boreal forest birds in southeastern Norway. *Annales Zoologici Fennici*, **49**, 69-80.

Healy, S.D. & Rowe, C. (2007) A critique of comparative studies of brain size. *Proceeding of the Royal Society of London. Series B, Biological Sciences*, **274**, 453-464.

Mlikovsky, J. (1989a) Brain size in birds: 2. Falconiformes through Gaviiformes. *Vestnik Ceskoslovenska Spolecnosti Zoologicka*, **53**, 200-213.

Mlikovsky, J. (1989b) Brain size in birds: 3. Columbiformes through Piciformes. *Vestnik Ceskoslovenska Spolecnosti Zoologicka*, **53**, 252-264.

Mlikovsky, J. (1990) Brain size in birds: 4. Passeriformes. *Acta Societatis Zoologicka Bohemoslovacae*, 54, 27-37.

Møller, A.P., Erritzøe, J. & Garamszegi, L.Z. (2005) Covariation between brain size and immunity in birds: implications for brain size evolution. *Journal of Evolutionary Biology*, **18**, 223-237.

Table S5. Associations between the frequency of occurrence of bird species (**forest species only, *N* = 62**) in urban sites in Oslo and six predictor variables (frequency in surrounding forest sites, habitat type, migration, nest site, (ln-transformed) brain mass and residual body mass); *Sequential regression used due to collinearity (i.e. VIF > 10) between body mass and brain mass. (a) results of full PGLS model. The model including the maximum-likelihood value of λ was compared against models including λ = 0 and 1; superscripts following λ estimates indicate p-values of the likelihood-ratio tests (first position: against λ = 0; second position: against λ = 1). Effect sizes (partial *r*) and their noncentral 95% confidence intervals (LCL, lower confidence limit; UCL, upper confidence limit) were calculated for each urban occurrence-predictor variable pair. (b) Results of ANCOVA summarised with Type III (simultaneous) sum of squares to examine overall effect of each of the six predictor variables. Significant relationships are shown in bold.

| **a)** | **Predictor** | **Estimate** ± **SE** | ***t*_53_** | ***P*** | **Partial *r*** | **LCL-UCL** | **λ** |
| --- | --- | --- | --- | --- | --- | --- | --- |
|  | Frequency in surrounding forest sites | **0.640 ± 0.10** | **6.20** | **< 0.0001** | **0.65** | **0.47 – 0.76** | 0 ^1, <0.0001^ |
|  | Forest type - deciduous | **0.335 ± 0.08** | **4.05** | **0.0002** | **0.49** | **0.26 – 0.65** |  |
|  | Migration | 0.013 ± 0.10 | 0.13 | 0.90 | 0.02 | -0.24 – 0.28 |  |
|  | Nest – ground | -0.245 ± 0.15 | -1.66 | 0.10 | -0.22 | -0.45 – 0.05 |  |
|  | Nest – high | 0.022 ± 0.10 | 0.22 | 0.82 | 0.03 | -0.23 – 0.29 |  |
|  | Nest – low | -0.030 ± 0.13 | -0.22 | 0.83 | -0.03 | -0.29 – 0.23 |  |
|  | Brain mass | -0.050 ± 0.05 | -1.00 | 0.32 | -0.14 | -0.38 – 0.13 |  |
|  | Residual body mass* | 0.131 ± 0.09 | 1.55 | 0.13 | 0.21 | -0.06 – 0.44 |  |

| **b)** | **Predictor** | ***df*** | ***F*** | ***P*** |
| --- | --- | --- | --- | --- |
|  | Frequency in surrounding forest sites | 1 | **38.41** | **< 0.0001** |
|  | Forest type | 1 | **16.41** | **0.0002** |
|  | Migration | 1 | 0.02 | 0.90 |
|  | Nest site | 3 | 1.41 | 0.25 |
|  | Brain mass | 1 | 1.00 | 0.32 |
|  | Residual body mass* | 1 | 2.40 | 0.13 |

Table S6. Associations between the frequency of occurrence of bird species (**farmland species only, *N* = 26**) in urban sites in Oslo and six predictor variables (frequency in surrounding farmland sites, habitat type, migration, nest site, (ln-transformed) brain mass and residual body mass). (a) results of full PGLS model. The model including the maximum-likelihood value of λ was compared against models including λ = 0 and 1; superscripts following λ estimates indicate p-values of the likelihood-ratio tests (first position: against λ = 0; second position: against λ = 1). Effect sizes (partial *r*) and their noncentral 95% confidence intervals (LCL, lower confidence limit; UCL, upper confidence limit) were calculated for each urban occurrence-predictor variable pair. (b) Results of ANCOVA summarised with Type III (simultaneous) sum of squares to examine overall effect of each of the six predictor variables. Significant relationships are shown in bold. Multicollinearity was not problematic in these models (i.e. all VIF < 7.88).

| **a)** | **Predictor** | **Estimate** ± **SE** | ***t_18_*** | ***P*** | **Partial *r*** | **LCL-UCL** | **λ** |
| --- | --- | --- | --- | --- | --- | --- | --- |
|  | Frequency in surrounding farmland sites | **0.872 ± 0.14** | **6.12** | **< 0.0001** | **0.82** | **0.61 – 0.90** | 0 ^1, 0.019^ |
|  | Migration | 0.061 ± 0.11 | 0.54 | 0.60 | 0.13 | -0.32 – 0.51 |  |
|  | Nest – ground | **-0.680 ± 0.14** | **-4.97** | **0.0001** | **-0.76** | **-0.87 – -0.49** |  |
|  | Nest – high | 0.122 ± 0.15 | 0.84 | 0.41 | 0.19 | -0.26 – 0.55 |  |
|  | Nest – low | **-0.358 ± 0.15** | **-2.42** | **0.03** | **-0.50** | **-0.73 – -0.06** |  |
|  | Brain mass | -0.239 ± 0.15 | -1.61 | 0.13 | -0.36 | -0.65 – 0.10 |  |
|  | Body mass | 0.181 ± 0.10 | 1.90 | 0.07 | 0.41 | -0.04 – 0.68 |  |

| **b)** | **Predictor** | ***df*** | ***F*** | ***P*** |
| --- | --- | --- | --- | --- |
|  | Frequency in surrounding farmland sites | 1 | **37.47** | **< 0.0001** |
|  | Migration | 1 | 0.29 | 0.60 |
|  | Nest site | 3 | **11.12** | **0.0002** |
|  | Brain mass | 1 | 2.58 | 0.13 |
|  | Body mass | 1 | 3.61 | 0.07 |

Table S7. Interspecific associations (controlling for phylogeny) between the frequency of occurrence of bird species (**forest species, *N* = 39**) in urban sites in Oslo and six predictor variables (**abundance in surrounding forests based on point counts published by Haavik and Dale (2012)**, habitat type, migration, nest site, (ln-transformed) brain mass and residual body mass). The model including the maximum-likelihood value of λ was compared against models including λ = 0 and 1; superscripts following λ estimates indicate p-values of the likelihood-ratio tests (first position: against λ = 0; second position: against λ = 1). Effect sizes (partial *r*) and their noncentral 95% confidence intervals (LCL, lower confidence limit; UCL, upper confidence limit) were calculated for each urban occurrence-predictor variable pair. Significant relationships are shown in bold.

| **Predictor** | **Estimate** ± **SE** | ***t_29_*** | ***P*** | **Partial *r*** | **LCL-UCL** | **λ** |
| --- | --- | --- | --- | --- | --- | --- |
| Abundance in surrounding forest | **0.002 ± 0.001** | **3.28** | **0.003** | **0.52** | **0.20 – 0-71** | 0.97 ^0.04, 0.84^ |
| Habitat – mixed/deciduous | **0.659 ± 0.12** | **5.71** | **< 0.0001** | **0.73** | **0.52 – 0.83** |  |
| Habitat – farmland | **0.913 ± 0.21** | **4.30** | **0.0002** | **0.62** | **0.35 – 0.77** |  |
| Migration | 0.135 ± 0.19 | 0.70 | 0.49 | 0.13 | -0.23 – 0.44 |  |
| Nest – ground | -0.164 ± 0.21 | -0.77 | 0.45 | -0.14 | -0.45 – 0.22 |  |
| Nest – high | 0.148 ± 0.22 | 0.67 | 0.51 | 0.12 | -0.24 – 0.44 |  |
| Nest – low | -0.017 ± 0.20 | -0.08 | 0.93 | -0.02 | -0.35 – 0.33 |  |
| Brain mass | 0.045 ± 0.10 | 0.45 | 0.66 | 0.08 | -0.27 – 0.41 |  |
| Residual body mass | 0.016 ± 0.17 | 0.10 | 0.92 | 0.02 | -0.33 – 0.36 |  |

Table S8. Associations between the frequency of occurrence of bird species (**full sample, *N* = 90**) in urban sites in Oslo and six predictor variables (**national population size estimate**, habitat type, migration, nest site, (ln-transformed) brain mass and residual body mass); * Sequential regression used as VIF > 10 (10.62, 11.41 body mass and brain mass respectively) (a) Results of full PGLS model. The model including the maximum-likelihood value of λ was compared against models including λ = 0 and 1; superscripts following λ estimates indicate p-values of the likelihood-ratio tests (first position: against λ = 0; second position: against λ = 1). Effect sizes (partial *r*) and their noncentral 95% confidence intervals (LCL, lower confidence limit; UCL, upper confidence limit) were calculated for each urban occurrence-predictor variable pair. (b) Results of ANCOVA summarised with Type III (simultaneous) sum of squares to examine overall effect of each of the six predictor variables. Significant relationships are shown in bold.

| a) | **Predictor** | **Estimate** ± **SE** | ***t_79_*** | ***P*** | **Partial *r*** | **LCL-UCL** | **λ** |
| --- | --- | --- | --- | --- | --- | --- | --- |
|  | Abundance in surrounding sites | **< 0.0001** | **4.33** | **< 0.0001** | **0.44** | **0.25 – 0.58** | 0 ^1, <0.0001^ |
|  | Habitat – mixed/deciduous | **0.404 ± 0.11** | **3.73** | **0.0004** | **0.39** | **0.19 – 0.54** |  |
|  | Habitat – farmland | **0.559 ± 0.11** | **4.95** | **< 0.0001** | **0.49** | **0.30 – 0.62** |  |
|  | Habitat – urban | 0.259 ± 0.20 | 1.29 | 0.20 | 0.14 | -0.08 – 0.34 |  |
|  | Migration | 0.176 ± 0.106 | 1.66 | 0.10 | 0.18 | -0.04 – 0.38 |  |
|  | Nest – ground | **-0.486 ± 0.15** | **-3.29** | **0.002** | **-0.35** | **-0.51 – -0.14** |  |
|  | Nest – high | 0.015 ± 0.11 | 0.14 | 0.89 | 0.02 | -0.20 – 0.23 |  |
|  | Nest – low | -0.089 ± 0.14 | -0.64 | 0.53 | -0.07 | -0.28 – 0.15 |  |
|  | Brain mass | -0.079 ± 0.06 | -1.44 | 0.15 | -0.16 | -0.36 – 0.06 |  |
|  | Residual body mass* | 0.131 ± 0.09 | 1.46 | 0.15 | 0.16 | -0.06 – 0.36 |  |

| **b)** | **Predictor** | ***df*** | ***F*** | ***P*** |
| --- | --- | --- | --- | --- |
|  | Abundance in surrounding sites | 1 | **18.75** | **< 0.0001** |
|  | Habitat | 3 | **8.81** | **< 0.0001** |
|  | Migration | 1 | 2.77 | 0.10 |
|  | Nest site | 3 | **4.52** | **0.006** |
|  | Brain mass | 1 | 2.08 | 0.15 |
|  | Residual body mass* | 1 | 2.14 | 0.15 |

Table S9. Interspecific associations (controlling for phylogeny) between the frequency of occurrence of bird species (sample using **brain data from the major single source** [Mlikovsky 1989a,b, 1990], ***N* = 82**) in urban sites in **Oslo** and six predictor variables (frequency of occurrence in surrounding sites, habitat type, migration, nest site, (ln-transformed) brain mass and residual body mass); *Sequential regression used due to collinearity (i.e. VIF > 10, (10.9). (a) Results of full PGLS model. The model including the maximum-likelihood value of λ was compared against models including λ = 0 and 1; superscripts following λ estimates indicate p-values of the likelihood-ratio tests (first position: against λ = 0; second position: against λ = 1). Effect sizes (partial *r*) and their noncentral 95% confidence intervals (LCL, lower confidence limit; UCL, upper confidence limit) were calculated for each urban occurrence-predictor variable pair. (b) Results of ANCOVA summarised with Type III (simultaneous) sum of squares to examine overall effect of each of the six predictor variables. Significant relationships are shown in bold.

| **a)** | **Predictor** | **Estimate** ± **SE** | ***t_71_*** | ***P*** | **Partial *r*** | **LCL-UCL** | **λ** |
| --- | --- | --- | --- | --- | --- | --- | --- |
|  | Frequency in surrounding sites | **1.012 ± 0.08** | **12.21** | **< 0.0001** | **0.82** | **0.74 – 0.87** | 0 ^1,< 0.0001^ |
|  | Habitat – mixed/deciduous | **0.263 ± 0.07** | **3.56** | **0.0007** | **0.40** | **0.18 – 0.55** |  |
|  | Habitat – farmland | **0.551 ± 0.08** | **7.32** | **< 0.0001** | **0.66** | **0.51 – 0.75** |  |
|  | Habitat – urban | **0.703 ± 0.14** | **4.89** | **< 0.0001** | **0.50** | **0.31 – 0.64** |  |
|  | Migration | 0.105 ± 0.07 | 1.50 | 0.14 | 0.18 | -0.06 – 0.38 |  |
|  | Nest – ground | **-0.385 ± 0.10** | **-3.97** | **0.0002** | **-0.43** | **-0.58 – -0.22** |  |
|  | Nest – high | 0.004 ± 0.07 | 0.06 | 0.96 | 0.007 | -0.22 – 0.23 |  |
|  | Nest – low | -0.187 ± 0.10 | -1.84 | 0.07 | -0.21 | -0.41 – 0.02 |  |
|  | Brain mass | -0.002 ± 0.04 | -0.05 | 0.96 | -0.006 | -0.23 – 0.22 |  |
|  | Residual body mass* | 0.117 ± 0.06 | 1.99 | 0.05 | 0.23 | -0.0005 – 0.43 |  |

| **b)** | **Predictor** | ***df*** | ***F*** | ***P*** |
| --- | --- | --- | --- | --- |
|  | Frequency in surrounding sites | 1 | **149.10** | **< 0.0001** |
|  | Habitat | 3 | **21.18** | **< 0.0001** |
|  | Migration | 1 | 2.26 | 0.14 |
|  | Nest site | 3 | **6.46** | **0.0006** |
|  | Brain mass | 1 | 0.002 | 0.96 |
|  | Residual body mass* | 1 | **4.00** | **0.05** |

Table S10. Interspecific associations (controlling for phylogeny) between the frequency of occurrence of bird species (sample using **brain data from Møller et al. 2005**, ***N* = 59** in urban sites in **Oslo** and six predictor variables ((frequency of occurrence in surrounding sites, habitat type, migration, nest site, (ln-transformed) brain mass and residual body mass); * Sequential regression used due to collinearity (i.e. VIF > 15.98). (a) Results of full PGLS model. The model including the maximum-likelihood value of λ was compared against models including λ = 0 and 1; superscripts following λ estimates indicate p-values of the likelihood-ratio tests (first position: against λ = 0; second position: against λ = 1). Effect sizes (partial *r*) and their noncentral 95% confidence intervals (LCL, lower confidence limit; UCL, upper confidence limit) were calculated for each urban occurrence-predictor variable pair. (b) Results of ANCOVA summarised with Type III (simultaneous) sum of squares to examine overall effect of each of the six predictor variables. Significant relationships are shown in bold.

| **a)** | **Predictor** | **Estimate** ± **SE** | ***t_48_*** | ***P*** | **Partial *r*** | **LCL-UCL** | **λ** |
| --- | --- | --- | --- | --- | --- | --- | --- |
|  | Frequency in surrounding sites | **1.043 ± 0.12** | **9.00** | **< 0.0001** | **0.79** | **0.67 – 0.86** | 0 ^1,< 0.0001^ |
|  | Habitat – mixed/deciduous | **0.276 ± 0.11** | **2.54** | **0.01** | **0.34** | **0.07 – 0.55** |  |
|  | Habitat – farmland | **0.572 ± 0.11** | **5.41** | **< 0.0001** | **0.62** | **0.41 – 0.74** |  |
|  | Habitat – urban | **0.689 ± 0.19** | **3.68** | **0.0006** | **0.47** | **0.22 – 0.64** |  |
|  | Migration | 0.054 ± 0.09 | 0.58 | 0.57 | 0.08 | -0.20 – 0.34 |  |
|  | Nest – ground | **-0.495 ± 0.14** | **-3.67** | **0.0006** | **-0.47** | **-0.64 – -0.22** |  |
|  | Nest – high | -0.002 ± 0.10 | -0.02 | 0.98 | -0.003 | -0.28 – 0.27 |  |
|  | Nest – low | -0.213 ± 0.11 | -1.86 | 0.07 | -0.26 | -0.49 – 0.02 |  |
|  | Brain mass | -0.022 ± 0.05 | -0.48 | 0.63 | -0.07 | -0.33 – 0.21 |  |
|  | Residual body mass* | 0.085 ± 0.10 | 0.83 | 0.41 | 0.12 | -0.16 – 0.37 |  |

| **b)** | **Predictor** | ***df*** | ***F*** | ***P*** |
| --- | --- | --- | --- | --- |
|  | Frequency in surrounding sites | 1 | **80.99** | **< 0.0001** |
|  | Habitat | 3 | **11.21** | **< 0.0001** |
|  | Migration | 1 | 0.33 | 0.57 |
|  | Nest site | 3 | **5.44** | **0.003** |
|  | Brain mass | 1 | 0.23 | 0.63 |
|  | Residual body mass* | 1 | 0.70 | 0.41 |

**Analysis of data from other European cities**

Data were from six European cities:

Heinola, Finland Vauhkonen (1990, table 1)

Angers, France Rose (2004, appendix 2)

Rennes, France Rose (2004, appendix 2)

Livorno, Italy Clergeau et al. (2006, table 2)

Pisa, Italy Clergeau et al. (2006, table 2)

Madrid, Spain Palomino and Carrascal (2006, table 2)

All papers reported data on bird communities in both urban and rural areas. For each study, there were data from 2-3 different urban zones: city centre, city suburbs and (for two cities only) other urban areas (e.g. parks only). In contrast to the data from Oslo, these sources reported species densities and not presence/absence data. Vauhkonen (1984) reported densities (pairs per km^2^) and Rose (2004) presented mean number of individuals per site. Clergeau et al. (2006) reported relative densities as % of all pairs recorded for passerine species, but their table 2 covered only the 29 most common species out of 65 species recorded. Palomino and Carrascal (2006) presented bird densities in three urban zones and four types of natural areas. We calculated an aggregate density estimate for the four natural areas by weighting according to the number of sample plots in each, thus giving a mean density across sample plots.

Clergeau, P., Croci, S., Jokimäki, J., Kaisanlahti-Jokimäki, M.-L. & Dinetti, M. (2006) Avifauna homogenisation by urbanisation: analysis at different European latitudes. *Biological Conservation*, **127**, 336-344.

Palomino, D. & Carrascal, L.M. (2006) Urban influence on birds at a regional scale: A case study with the avifauna of northern Madrid province. *Landscape and Urban Planning*, **77**, 276-290.

Rose, F. (2004) *Caractérisation de l'avifaune le long d'un gradient d'urbanisation à Rennes et Angers*. Report, INRA, Rennes, France.

Vauhkonen, M. (1990) Heinolan kaupunkialueen pesimälinnusto vuosina 1989-1990. *Päijät-Hämeen linnut*, **21**, 112-121.

Table S11. Land bird species reported in previously published studies from six European cities (species in bold lacked brain data and were not included in the present study):

Species Heinola Angers Rennes Livorno Pisa Madrid

________________________________________________________________________________________________________________________

*Apus apus X X*

*Streptopelia decaocto X X X*

*Streptopelia turtur X X*

*Columba livia X X X*

*Columba palumbus X X X*

*Ciconia ciconia X*

*Cuculus canorus X X X*

*Buteo buteo X X*

*Upupa epops X*

*Merops apiaster X*

*Jynx torquilla X*

*Dendrocopos major X X X X*

*Dendrocopos minor X X*

*Picus viridis X X X*

*Oriolus oriolus X*

*Cyanopica cyana X*

*Garrulus glandarius X X X*

*Pica pica X X X X X X*

*Corvus monedula X X X X X*

*Corvus corone X X X X X X*

*Cyanistes caeruleus X X X X X X*

*Parus major X X X X X X*

*Lophophanes cristatus X X X X*

*Periparus ater X X X*

*Poecile palustris X X*

*Poecile montanus X*

*Alauda arvensis X*

***Lullula arborea*** *X X*

*Locustella naevia X*

Species Heinola Angers Rennes Livorno Pisa Madrid

________________________________________________________________________________________________________________________

*Acrocephalus palustris X*

***Acrocephalus dumetorum*** *X*

*Hippolais icterina X*

***Hippolais polyglotta*** *X*

*Sylvia atricapilla X X X X X X*

*Sylvia borin X*

*Sylvia curruca X*

***Sylvia cantillans*** *X*

*Sylvia communis X*

*Delichon urbica X X X X*

*Hirundo rustica X X X X X X*

*Aegithalos caudatus X X X*

***Cettia cetti*** *X*

*Phylloscopus bonelli X*

*Phylloscopus sibilatrix X*

*Phylloscopus collybita X X X*

*Phylloscopus trochilus X X X*

*Regulus regulus X X*

***Regulus ignicapillus*** *X X*

*Sitta europaea X X X*

*Troglodytes troglodytes X X X X X*

*Certhia brachydactyla X X X X X*

*Sturnus vulgaris X X X X X*

***Sturnus unicolor*** *X*

*Turdus viscivorus X X X*

*Turdus philomelos X X X*

*Turdus iliacus X*

*Turdus merula X X X X X X*

*Turdus pilaris X*

*Muscicapa striata X X*

*Erithacus rubecula X X X X X X*

***Luscinia megarhynchos*** *X*

Species Heinola Angers Rennes Livorno Pisa Madrid

________________________________________________________________________________________________________________________

*Luscinia luscinia X*

*Ficedula hypoleuca X X*

*Phoenicurus phoenicurus X*

*Phoenicurus ochruros X*

*Oenanthe oenanthe X*

*Saxicola torquata X*

*Saxicola rubetra X*

*Prunella modularis X X*

*Passer domesticus X X X X X X*

*Passer montanus X X X X*

*Anthus trivialis X X X*

*Motacilla alba X X X X X*

*Emberiza cirlus X*

*Emberiza citrinella X*

*Miliaria calandria X*

*Fringilla coelebs X X X X X X*

*Coccohtraustes coccothraustes X*

*Carpodacus erythrinus X*

*Chloris chloris X X X X X X*

*Carduelis cannabina X X X X*

*Loxia curvirostra X*

*Carduelis carduelis X X X*

*Carduelis spinus X*

*Serinus serinus X X X X X*

________________________________________________________________________________________________________________________Table S12. Interspecific associations (controlling for phylogeny) between the relative density of bird species (***N* = 20**) in urban zones in **Livorno** and three predictor variables: relative density outside city, (ln-transformed) brain mass and residual body mass. * Sequential regression used due to collinearity (i.e. VIF >10). The model including the maximum-likelihood value of λ was compared against models including λ = 0 and 1; superscripts following λ estimates indicate p-values of the likelihood-ratio tests (first position: against λ = 0; second position: against λ = 1). Effect sizes (partial *r*) and their noncentral 95% confidence intervals (LCL, lower confidence limit; UCL, upper confidence limit) were calculated for each urban occurrence-predictor variable pair. Significant relationships are shown in bold.

________________________________________________________________________________________________________________________

**Predictor slope *t_16_ P* λ Partial *r* LCL – UCL**

________________________________________________________________________________________________________________________

*a) City centre*

Relative density outside city **1.13 7.93 < 0.0001** 0 ^1, 0.0007^ **0.89 0.75 – 0.94**

Residual body mass* -0.06 -0.69 0.50 -0.17 -0.55 – 0.31

Brain mass 0.04 1.32 0.21 0.31 -0.18 – 0.64

*b) Suburbs*

Relative density outside city **1.07 14.36 < 0.0001** 0 ^1, 0.0008^ **0.96 0.91 – 0.98**

Residual body mass* 0.02 0.35 0.73 0.09 -0.37 – 0.42

Brain mass 0.02 1.22 0.24 0.29 -0.20 – 0.63

________________________________________________________________________________________________________________________Table S13. Interspecific associations (controlling for phylogeny) between the relative density of bird species (***N* = 20**) in urban zones in **Pisa** and three predictor variables: relative density outside city, (ln-transformed) brain mass and residual body mass. * Sequential regression used due to collinearity (i.e. VIF >10). The model including the maximum-likelihood value of λ was compared against models including λ = 0 and 1; superscripts following λ estimates indicate p-values of the likelihood-ratio tests (first position: against λ = 0; second position: against λ = 1). Effect sizes (partial *r*) and their noncentral 95% confidence intervals (LCL, lower confidence limit; UCL, upper confidence limit) were calculated for each urban occurrence-predictor variable pair. Significant relationships are shown in bold.

________________________________________________________________________________________________________________________

**Predictor slope *t_16_ P* λ Partial *r* LCL – UCL**

________________________________________________________________________________________________________________________

*a) City centre*

Relative density outside city **0.82 5.47 < 0.0001** 0 ^1, 0.001^ **0.81 0.56 – 0.90**

Residual body mass* 0.04 0.39 0.70 0.10 -0.37 – 0.51

Brain mass 0.01 0.44 0.67 0.11 -0.36 – 0.51

*b) Suburbs*

Relative density outside city **0.97 14.52 < 0.0001** 0 ^1, 0.002^ **0.96 0.92** – **0.98**

Residual body mass* 0.008 0.18 0.86 0.05 -0.41 – 0.47

Brain mass -0.014 -1.18 0.26 -0.28 -0.62 – 0.21

________________________________________________________________________________________________________________________Table S14. Interspecific associations (controlling for phylogeny) between the density of bird species (***N* = 46**) in urban zones in **Madrid** and three predictor variables (density outside city and relative brain mass [i.e. ln-transformed body mass and brain mass]). The model including the maximum-likelihood value of λ was compared against models including λ = 0 and 1; superscripts following λ estimates indicate p-values of the likelihood-ratio tests (first position: against λ = 0; second position: against λ = 1). Effect sizes (partial *r*) and their noncentral 95% confidence intervals (LCL, lower confidence limit; UCL, upper confidence limit) were calculated for each urban occurrence-predictor variable pair. Significant relationships are shown in bold.

________________________________________________________________________________________________________________________

**Predictor slope *t_42_ P* λ Partial *r* LCL – UCL**

________________________________________________________________________________________________________________________

*a) City centre*

Density outside city 0.24 0.82 0.42 0 ^1, < 0.0001^ 0.12 -0.18 – 0.39

Body mass 0.19 1.06 0.30 0.16 -0.14 – 0.42

Brain mass -0.31 -1.09 0.28 -0.17 -0.43 – 0.14

*b) Suburbs*

Density outside city **0.88 3.22 0.003** 0 ^1, < 0.0001^ **0.45 0.17 – 0.63**

Body mass 0.09 0.53 0.60 0.08 -0.22 – 0.36

Brain mass -0.17 -0.67 0.51 -0.10 -0.38 – 0.20

*b) Other urban areas*

Density outside city **0.60 2.52 0.02** 0 ^1, < 0.0001^ **0.36 0.07 – 0.57**

Body mass 0.09 0.59 0.56 0.09 -0.21 – 0.37

Brain mass -0.17 -0.74 0.46 -0.11 -0.39 – 0.19

________________________________________________________________________________________________________________________Table S15. Interspecific associations (controlling for phylogeny) between the density of bird species (***N* = 43**) in urban zones in **Angers** and three predictor variables (density outside city and relative brain mass [i.e. ln-transformed body mass and brain mass]). The model including the maximum-likelihood value of λ was compared against models including λ = 0 and 1; superscripts following λ estimates indicate p-values of the likelihood-ratio tests (first position: against λ = 0; second position: against λ = 1). Effect sizes (partial *r*) and their noncentral 95% confidence intervals (LCL, lower confidence limit; UCL, upper confidence limit) were calculated for each urban occurrence-predictor variable pair. Significant relationships are shown in bold.

________________________________________________________________________________________________________________________

**Predictor slope *t_39_ P* λ Partial *r* LCL – UCL**

________________________________________________________________________________________________________________________

*a) City centre*

Density outside city **0.93 10.83 < 0.0001** 0 ^1, < 0.0001^ **0.87 0.78 – 0.91**

Body mass 0.12 0.96 0.34 0.15 -0.16 – 0.42

Brain mass -0.05 -0.25 0.81 -0.04 -0.33 – 0.27

*b) Suburbs*

Density outside city **0.88 9.54 < 0.0001** 0 ^1, 0.0007^ **0.84 0.73 – 0.89**

Body mass -0.11 -1.12 0.27 -0.18 -0.44 – 0.14

Brain mass 0.20 1.24 0.22 0.20 -0.12 – 0.46

________________________________________________________________________________________________________________________Table S16. Interspecific associations (controlling for phylogeny) between the density of bird species (***N* = 33**) in urban zones in **Rennes** and three predictor variables (density outside city and relative brain mass [i.e. ln-transformed body mass and brain mass]). The model including the maximum-likelihood value of λ was compared against models including λ = 0 and 1; superscripts following λ estimates indicate p-values of the likelihood-ratio tests (first position: against λ = 0; second position: against λ = 1). Effect sizes (partial *r*) and their noncentral 95% confidence intervals (LCL, lower confidence limit; UCL, upper confidence limit) were calculated for each urban occurrence-predictor variable pair. Significant relationships are shown in bold.

________________________________________________________________________________________________________________________

**Predictor slope *t_29_ P* λ Partial *r* LCL – UCL**

________________________________________________________________________________________________________________________

*a) City centre*

Density outside city **0.98 7.12 < 0.0001** 0 ^1, 0.0003^ **0.80 0.63 – 0.88**

Body mass 0.09 0.68 0.50 0.13 -0.23 – 0.44

Brain mass -0.13 -0.60 0.55 -0.11 -0.43 – 0.25

*b) Suburbs*

Density outside city **1.03 11.01 < 0.0001** 0 ^1, 0.0009^ **0.90 0.81 – 0.94**

Body mass 0.03 0.27 0.79 0.05 -0.30 – 0.38

Brain mass 0.03 0.22 0.83 0.04 -0.31 – 0.38

________________________________________________________________________________________________________________________Table S17. Interspecific associations (controlling for phylogeny) between the density of bird species (***N* = 48**) in urban zones in **Heinola** and three predictor variables (density outside city and relative brain mass [i.e. ln-transformed body mass and brain mass]). The model including the maximum-likelihood value of λ was compared against models including λ = 0 and 1; superscripts following λ estimates indicate p-values of the likelihood-ratio tests (first position: against λ = 0; second position: against λ = 1). Effect sizes (partial *r*) and their noncentral 95% confidence intervals (LCL, lower confidence limit; UCL, upper confidence limit) were calculated for each urban occurrence-predictor variable pair. Significant relationships are shown in bold.

________________________________________________________________________________________________________________________

**Predictor slope *t_44_ P* λ Partial *r* LC – UCL**

________________________________________________________________________________________________________________________

*a) City centre*

Density outside city **1.05 3.73 0.0005** 0 ^1, < 0.0001^ **0.49 0.24 – 0.66**

Body mass 0.67 0.71 0.48 0.11 -0.19 – 0.37

Brain mass -0.67 -0.49 0.63 -0.07 -035 – 0.22

*b) Suburbs*

Density outside city **0.89 6.85 < 0.0001** 0 ^1, < 0.0001^ **0.72 0.55 – 0.81**

Body mass 0.27 0.62 0.54 0.09 -0.20 – 0.36

Brain mass -0.23 -0.37 0.72 -0.06 -0.33 – 0.23

*b) Other urban areas*

Density outside city **1.13 9.04 < 0.0001** 0 ^1, 0.0001^ **0.81 0.69 – 0.87**

Body mass -0.39 -0.94 0.35 -0.14 -0.40 – 0.15

Brain mass 0.77 1.27 0.21 0.19 -0.11 – 0.44

________________________________________________________________________________________________________________________
