# Supplementary material for: Commonness and ecology, but not bigger brains, predict urban living in birds
Source: BMC Ecol. 2015 Apr 11;15:12. doi: 10.1186/s12898-015-0044-x (PMC4412207; doi:10.1186/s12898-015-0044-x)

Additional file 2
Commonness and ecology, but not bigger brains, predict urban living in birds
Svein Dale, Jan T. Lifjeld and Melissah Rowe

Figure S1. Map of Oslo and urban study sites. Black patches within the thick solid line indicate urban study sites. Note that some sites were close to each other so that boundaries between all 93 sites are not visible on the map. The thick solid line marks the boundary between urban areas in Oslo and forest (Fo) and farmland areas (Fa). This boundary is abrupt because of strict enforcement of a ban on building houses outside the line (in Norwegian: Markagrensen; along the whole northern part of urban Oslo and along most of the eastern part). In some places urban parts of Oslo border to suburban areas (Su) of neighbouring municipalities. Stippled lines in Oslofjorden (fiord) indicate islands. Stippled lines within the urban area in Oslo indicate the three major green areas that were not included in the present study. Rural study sites were located to the west, north, north-east and south-east of the urban areas.

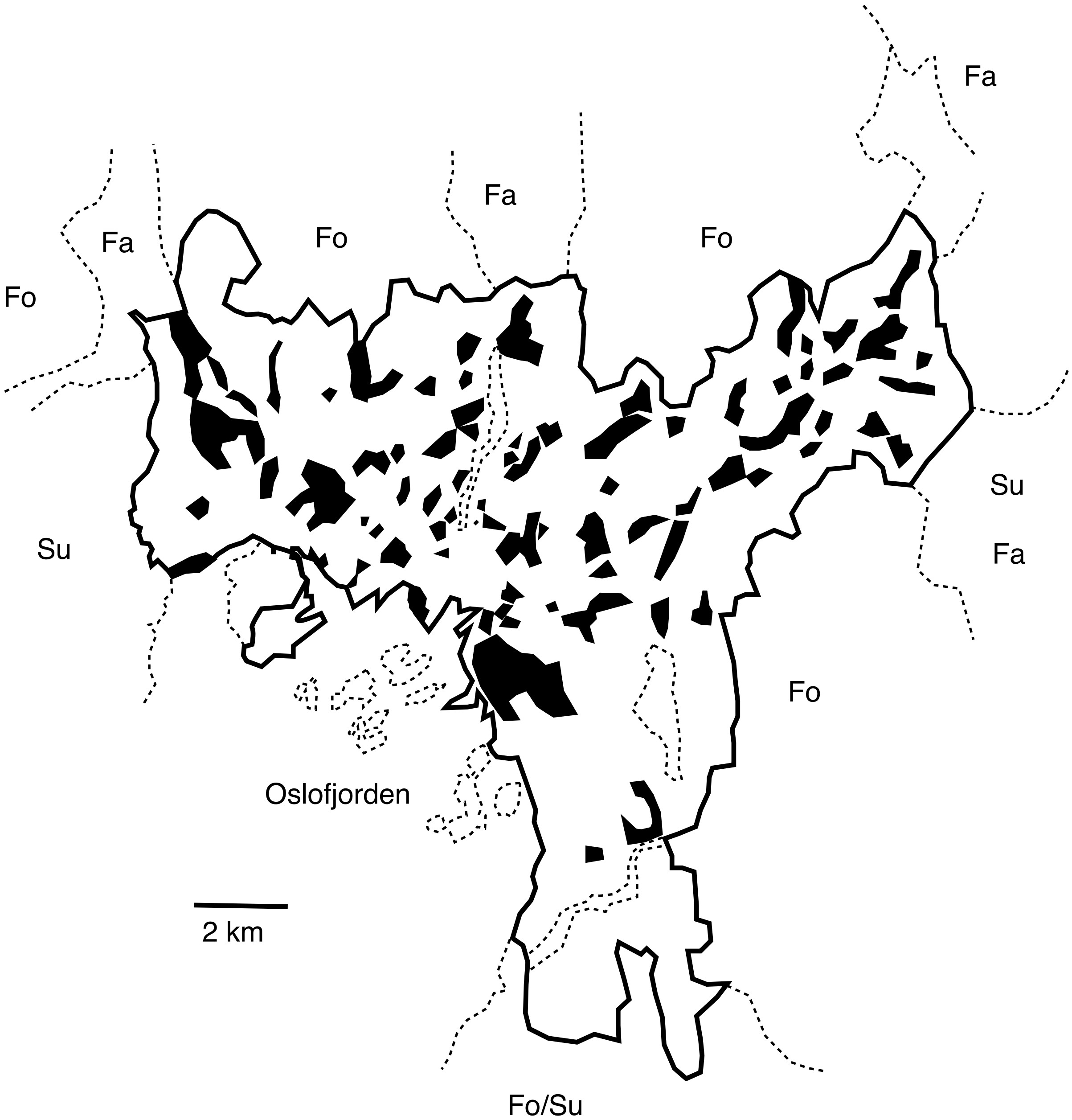

Supplement: Additional file 2: Figure S1. — Map of urban study sites in Oslo. [file 12898_2015_44_MOESM2_ESM.pdf]
