# Supplementary material for: Commonness and ecology, but not bigger brains, predict urban living in birds
Source: BMC Ecol. 2015 Apr 11;15:12. doi: 10.1186/s12898-015-0044-x (PMC4412207; doi:10.1186/s12898-015-0044-x)
Supplement: Additional file 3: — Supplementary methods. Evaluation of possible biases in data. [file 12898_2015_44_MOESM3_ESM.docx]

Additional file 3

Supplementary methods

Commonness and ecology, but not bigger brains, predict urban living in birds

Svein Dale, Jan T. Lifjeld and Melissah Rowe

Evaluation of possible biases in data

*Relative coverage of rural forest and farmland sites*

The number of forest and farmland sites (*N* = 137 and 51, respectively) in rural areas reflected to a large degree the relative availability of these habitats around Oslo; forest cover much larger areas than farmland (see Additional file 2: Figure S1), although the exact proportion depends on how far away from the city one defines potential source areas. However, any potential bias in the proportion used in the overall analyses is removed in the analyses made for forest and farmland bird species separately (Additional file 1: Tables S5-S6) because each of these analyses used frequencies in rural sites based on only one habitat (either rural forest sites or rural farmland sites).

*Effect of sampling effort in urban sites*

One could argue that sampling effort could affect our measure of relative commonness of species. We sampled urban sites three times, but we asked whether relative commonness would be different with e.g. lower sampling effort. To test this we compared the relative ranking of species after one round of censuses (randomly chosen from three rounds of censuses so that one third of sites had data from the census made early in the breeding season, one third from the middle of the breeding season, and one third from late in the breeding season) with the relative ranking after all three rounds of censuses was completed. There was a strong and positive correlation (*r_s_* = 0.98, n = 60, *P* < 0.0001; Figure SM1). This suggests that relative commonness of species is not affected by sampling effort. Furthermore, after one round of censuses the mean number of species per site was 16.4, after two rounds 20.2 species, and after three censuses 22.4 species, indicating that the marginal increase in species per round of census had already dropped considerably so that using three censuses per site captured a large proportion of species present.

*Variable number of surveys in rural sites*

Our analyses focused on relative measures of commonness of species, and we were not interested in absolute measures of density *per se*. Consequently, the variable number of surveys in rural sites (compared to three visits to each urban site) should not bias our measure of commonness. To test this assumption, we compared relative commonness among subsets of our rural data in relation to sampling effort (number of surveys performed). We did this comparison separately for farmland and forest species/sites. We classified sampling effort in two groups, those with lower sampling effort (1-2 surveys per site) and higher sampling effort (3 or more surveys per site). For both farmland and forest species/sites, there were strong positive correlations between the two independent data sets. For farmland the correlation between number of sites each species was present in the two data sets was highly significant (*r_s_* = 0.95, *N* = 26, *P* < 0.0001; Figure SM2A). The corresponding correlation for forest species was also highly significant (*r_s_* = 0.92, *N* = 62, *P* < 0.0001; Figure SM2D). Furthermore, ranking in each subset of the data was strongly correlated to overall ranking of commonness based on all data (*r_s_* = 0.97, *N* = 26, *P* < 0.0001, Figure SM2B; *r_s_* = 0.99, *N* = 26, *P* < 0.0001, Figure SM2C; *r_s_* = 0.99, *N* = 62, *P* < 0.0001, Figure SM2E; *r_s_* = 0.96, *N* = 62, *P* < 0.0001, Figure SM2F). These results indicate that the relative ranking of species' commonness did not depend on sampling effort and remained similar irrespective of whether data from sites with lower or higher sampling effort was used. Thus, we conclude that the overall measure of species commonness which we used in the main analyses was robust and unaffected by differences in the number of surveys made.

*Different length of survey periods*

The longer survey period (1995-2011) of rural sites relative to urban sites (2003-2007) could potentially introduce a bias in our data if the rural data contained species which had significantly different population sizes either before or after the urban data were collected. In particular, because of recent population declines of many species in Europe (Inger et al. 2014), species could potentially have been present only before we started collecting data in urban sites, and thus presence in rural sites in contrast to absence in urban sites would be an artefact of survey period. However, to our knowledge, none of the species included in our study showed any major changes in the Oslo region during the study period. This general and subjective opinion was supported by the fact that among the 90 species included in this study, only 12 (13%) were listed on the Norwegian Red List (Kålås et al. 2010), compared to 33% (77/232) of all species in Norway (χ^2^ = 14.93, *df* = 1, *P* < 0.0001). Furthermore, among the 12 red listed species, four were actually common or fairly common within the urban areas (*Apus apus*, *Streptopelia decaocto*, *Sturnus vulgaris*, *Carduelis cannabina*). The remaining eight species which were mostly absent from urban sites (*Coturnix coturnix*, *Crex crex*, *Vanellus vanellus*, *Pernis apivorus*, *Accipiter gentilis*, *Lanius collurio*, *Alauda arvensis*, *Carpodacus erythrinus*) were to a large degree recorded concurrently with the field work in urban sites. Of a total of 203 records (one record was one of the eight species observed in one site in one year), 90 (44%) were made during 2003-2007 when urban sites were censused, 53 (26%) before that period, and 60 (30%) after. This was due to a generally larger survey effort in rural sites after 2005. This suggests that temporal biases were unlikely to influence our data much because a substantial part of rural data were collected during a short period which overlapped with urban censuses. Furthermore, the eight individual species did not show significant temporal changes in occurrence pattern in the rural sites (Spearman rank correlations of number of records in relation to year, *P* > 0.10 for all except *Vanellus vanellus* which had an increasing number of records, *P* = 0.04). Thus, we conclude that it is unlikely that our data was biased due to population changes in rural areas.

*Detectability of species in rural and urban sites*

Another potential source of bias could be that rural habitats differed from urban habitats in species detectability. We cannot exclude the possibility that detectability differed to some degree, but note that data were collected during the main breeding period when the majority of species sing actively to defend territories, and habitat differences has little impact on vocal detection. Furthermore, because we were interested in relative commonness in rural versus urban sites, an average difference in detectability across species between rural and urban sites would not matter; the relative order of species' commonness within rural versus urban sites is what matters. Note also that occurrence frequencies (occupancy rates) of our species spanned a range from 0-100% of sites, thus, even if there were some differences between species in their relative detectability in rural versus urban habitats, this is unlikely to substantially change either the relative order or the absolute values of occurrence frequencies of species. Species detectability should be most different between farmland and forest, and if the independent tests of these two source areas give similar results this would suggest that our conclusions are robust to potential differences in detectability.

*Representativeness of urban and rural sites*

One could argue that biases might appear in our measures of commonness if species have a patchy distribution so that neighbouring sites may share some species more often than with more distant sites. This could create a bias if our selection of sites is clumped so that some species may have been missed and some have been oversampled. However, for the urban sites we selected nearly all urban green spaces and they were fairly well spread out across the city (see Additional file 2: Figure S1). Rural sites were deliberately chosen to be spread out over large areas (distributed across approx. 800 km^2^), and we think it is unlikely that patchy distribution of species has biased our measures of commonness.

References

Inger, R., R. Gregory, J. P. Duffy, I. Stott, P. Voříšek, K. J. Gaston, and J. Hill. 2015. Common European birds are declining rapidly while less abundant species' numbers are rising. Ecology Letters 18:28-36.

Kålås, J. A., Å. Viken, S. Henriksen, and S. Skjelseth, eds. 2010. The 2010 Norwegian Red List for Species. Norwegian Biodiversity Information Centre, Trondheim, Norway.

Legends to Supplementary methods figures

Figure SM1. Correlation between number of sites each species (*N* = 60) was present after a single round of censuses in urban sites (*N* = 93) and after three rounds of censuses of urban sites was completed.

Figure SM2. Correlations between number of sites each species was present for different samples of rural sites with different sampling intensity.

A-C: Farmland species (*N* = 26).

A: Sites censused 1-2 times (*N* = 25) versus sites censused 3 or more times (*N* = 26).

B: Sites censused 1-2 times (*N* = 25) versus all sites combined (*N* = 51).

C: Sites censused 3 or more times (*N* = 26) versus all sites combined (*N* = 51).

D-F: Forest species (*N* = 62).

D: Sites censused 1-2 times (*N* = 95) versus sites censused 3 or more times (*N* = 42).

E: Sites censused 1-2 times (*N* = 95) versus all sites combined (*N* = 137).

F: Sites censused 3 or more times (*N* = 42) versus all sites combined (*N* = 137).
