# Supplementary material for: Commonness and ecology, but not bigger brains, predict urban living in birds
Source: BMC Ecol. 2015 Apr 11;15:12. doi: 10.1186/s12898-015-0044-x (PMC4412207; doi:10.1186/s12898-015-0044-x)

Additional file 4

Commonness and ecology, but not bigger brains, predict urban living in birds

Svein Dale, Jan T. Lifjeld and Melissah Rowe

Figure S2. Maximum clade credibility tree illustrating the evolutionary relationships of the 90 species included in the study of urbanization in Oslo (see main text for details of tree construction).

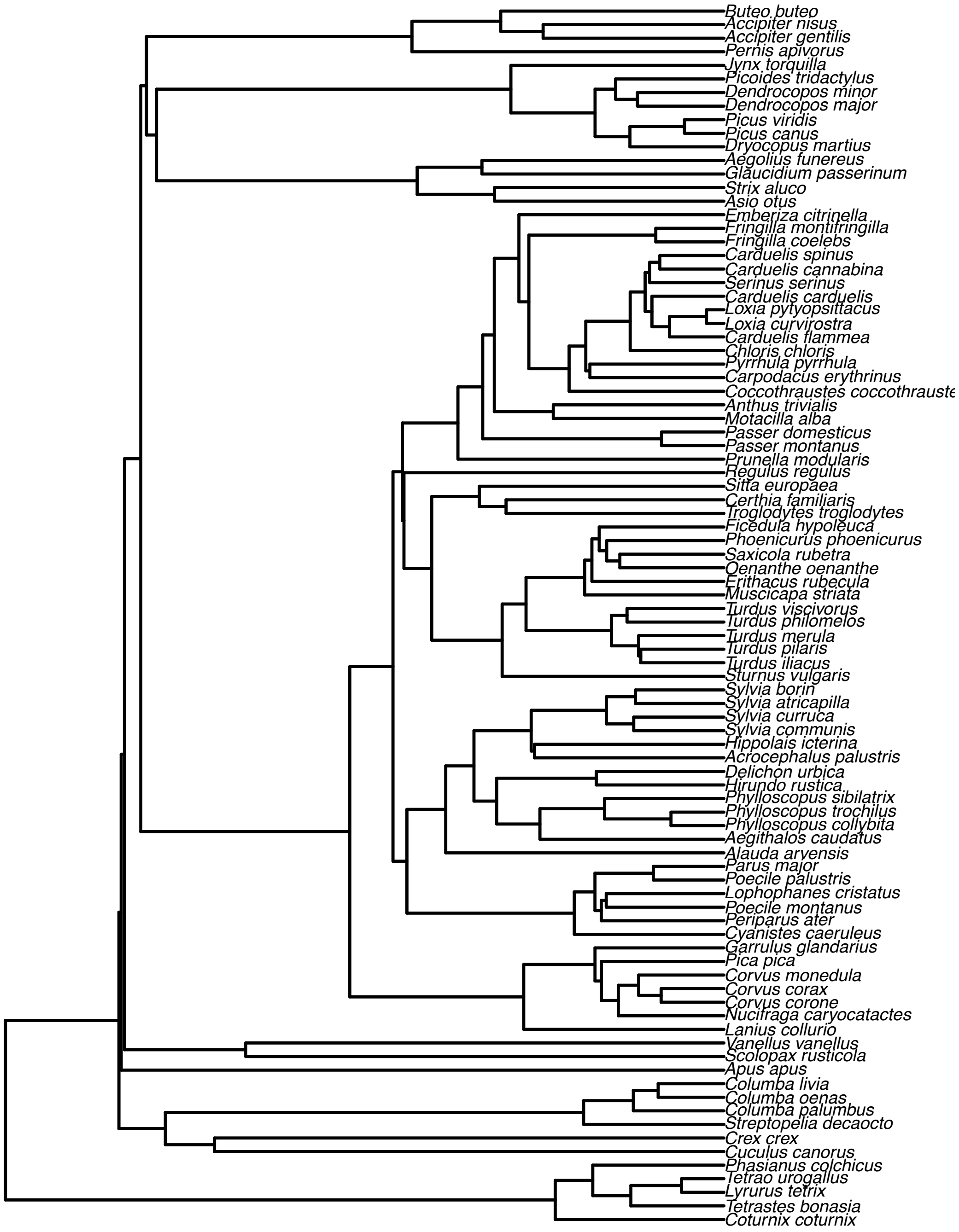

Supplement: Additional file 4: Figure S2. — Evolutionary relationships of the 90 species from Oslo. [file 12898_2015_44_MOESM4_ESM.pdf]
